# Synaptic vesicle glycoprotein 2 A in serum is an ideal biomarker for early diagnosis of Alzheimer’s disease

**DOI:** 10.1186/s13195-024-01440-9

**Published:** 2024-04-13

**Authors:** Xiaoling Wang, Xiaomin Zhang, Jing Liu, Jingjing Zhang, Congcong Liu, Yuting Cui, Qiao Song, Yuli Hou, Yaqi Wang, Qian Zhang, Yingzhen Zhang, Yujian Fan, Jianping Jia, Peichang Wang

**Affiliations:** 1 Department of Clinical Laboratory, Xuanwu Hospital, National Clinical Research Center for Geriatric Diseases, Capital Medical University, 45 Changchun Street, Beijing, 100053 China; 2 National Clinical Research Center for Geriatric Disorders, 45 Changchun Street, Beijing, 100053 China; 3 Innovation Center for Neurological Disorders, Department of Neurology, Xuanwu Hospital, Capital Medical University, 45 Changchun Street, Beijing, 100053 China; 4 Beijing Key Laboratory of Geriatric Cognitive Disorders, 45 Changchun Street, Beijing, 100053 China; 5 Clinical Center for Neurodegenerative Disease and Memory Impairment, Capital Medical University, 45 Changchun Street, Beijing, 100053 China; 6 Center of Alzheimer’s Disease, Beijing Institute for Brain Disorders, 45 Changchun Street, Beijing, 100053 China; 7 Key Laboratory of Neurodegenerative Diseases, Ministry of Education, 45 Changchun Street, Beijing, 100053 China

**Keywords:** Alzheimer’s disease, Serum SV2A, *APOE* ε4 carriers, Early diagnosis, Simoa

## Abstract

**Background:**

Previous studies have demonstrated that early intervention was the best plan to inhibit the progression of Alzheimer’s disease (AD), which relied on the discovery of early diagnostic biomarkers. In this study, synaptic vesicle glycoprotein 2 A (SV2A) was examined to improve the early diagnostic efficiency in AD.

**Methods:**

In this study, biomarker testing was performed through the single-molecule array (Simoa). A total of 121 subjects including cognitively unimpaired controls, amnestic mild cognitive impairment (aMCI), AD and other types of dementia underwent cerebrospinal fluid (CSF) SV2A testing; 430 subjects including health controls, aMCI, AD and other types of dementia underwent serum SV2A, glial fibrillary acidic protein (GFAP), neurofilament light chain (NfL) and p-tau217 testing; 92 subjects including aMCI and AD underwent both CSF SV2A and serum SV2A testing; 115 cognitively unimpaired subjects including *APOE* ε4 carriers and *APOE* ε4 non-carriers were tested for serum SV2A, GFAP, NfL and p-tau217. Then, the efficacy of SV2A for the early diagnosis of AD and its ability to identify those at high risk of AD from a cognitively unimpaired population were further analyzed.

**Results:**

Both CSF and serum SV2A significantly and positively correlated with cognitive performance in patients with AD, and their levels gradually decreased with the progression of AD. Serum SV2A demonstrated excellent diagnostic efficacy for aMCI, with a sensitivity of 97.8%, which was significantly higher than those of NfL, GFAP, and p-tau217. The SV2A-positive rates ranged from 92.86 to 100% in aMCI cases that were negative for the above three biomarkers. Importantly, of all the biomarkers tested, serum SV2A had the highest positivity rate (81.82%) in individuals at risk for AD.

**Conclusions:**

Serum SV2A was demonstrated to be a novel and ideal biomarker for the early diagnosis of AD, which can effectively distinguish those at high risk of AD in cognitively unimpaired populations.

**Supplementary Information:**

The online version contains supplementary material available at 10.1186/s13195-024-01440-9.

## Background

Growing evidence indicated that patients suffering from Alzheimer’s disease (AD) had begun to experience pathological changes and brain damage decades before the emergence of the first symptoms [1–3]. Therefore, any disease-modifying treatment will only be effective if treatment is started in the early stages of AD, which relies on the warning and early diagnosis [4]. However, no effective early warning and diagnostic blood biomarkers have been established, although biomarkers such as Aβ42/Aβ40 [5], phosphorylated tau (p-tau) [6, 7], neurofilament light (NfL) [8], and glial fibrillary acidic protein (GFAP) [9, 10] have demonstrated some efficacy in the early diagnosis of AD.

Postmortem studies have revealed consistently lower synapse numbers in the hippocampus and cortex of the AD group than those of the control group [11], and synaptic loss was recognized as the early pathological manifestation of AD [12, 13]. Synaptic vesicle glycoprotein 2 A (SV2A), located in the synaptic vesicles at presynaptic terminals, is regarded as the first in vivo marker of synaptic density [14], and numerous studies have used synaptic positron emission tomography (PET) tracers (e.g., [^11^C]UCB-J [15, 16] and [^18^F]UCB-H [17, 18]) to indicate the SV2A level in the hippocampal tissue of the participants to identify those with early AD and assess responses to disease-modifying therapy. Another study concluded that levetiracetam, with SV2A as the binding target [19], might help ameliorate related abnormalities in people who have or are at risk for AD [20]. Briefly, the above studies have indicated that SV2A has the potential to be an early diagnostic biomarker and therapeutic target for AD. Unfortunately, no studies have reported the role of SV2A in the blood for the early diagnosis of AD.

In this study, SV2A levels in serum and CSF at different stages of AD and other types of dementia were analyzed using single-molecule array (Simoa) technology. In addition, other well-studied AD biomarkers, including NfL, GFAP, and p-tau217, were detected in the serum of the above diagnostic groups to compare their efficacy with SV2A as an early diagnosis marker of AD.

## Methods

### Study participants

A total of 757 participants were recruited from September 2021 to February 2024. The diagnoses of AD was made according to the criteria published by the National Institute on Aging and Alzheimer’s Association (NIA-AA) [21], which combined with brain MRI or/and PET-Aβ or/and PET-tau. aMCI was diagnosed by the criteria as described previously [22]. Briefly, (I) cognitive impairment is detected by the patients themselves, their families, or experienced clinicians; (II) cognitive assessment reveals the presence of impairment in one or more domains of cognitive functioning; (III) complex instrumental daily living of patients can be mildly impaired but independent daily living is maintained; (IV) criteria for the diagnosis of dementia has not been met. VaD [23] and PDD [24] was diagnosed based on previously published criteria. All clinical diagnoses were first time diagnoses and were made by experienced physicians of Department of Neurology, Xuanwu Hospital, Capital Medical University.

The exclusion criteria for the patients was as follows: (I) patients with traumatic brain disorders and other psychiatric disorders; (II) patients with systemic diseases such as tumors, blood diseases, hypertension, and hyperglycemia. The inclusion criteria for the healthy controls for blood tests was as follows: (I) age-matched participants who have undergone medical examination at the Health Examination Center of Xuanwu Hospital, and (II) the results of the physical examination confirmed that the participants had no neurological diseases, hypertension, hyperglycemia, and other systemic diseases. The inclusion criteria for the participants in the control group who underwent CSF testing was as follows: (I) participants who were hospitalized for the first time and did not receive systemic therapy, (II) did not have imaging findings of cognitive impairment such as hippocampal atrophy, (III) and at least two neurologists confirmed that they did not have clinical manifestations of cognitive impairment.

**Fig. 1:**
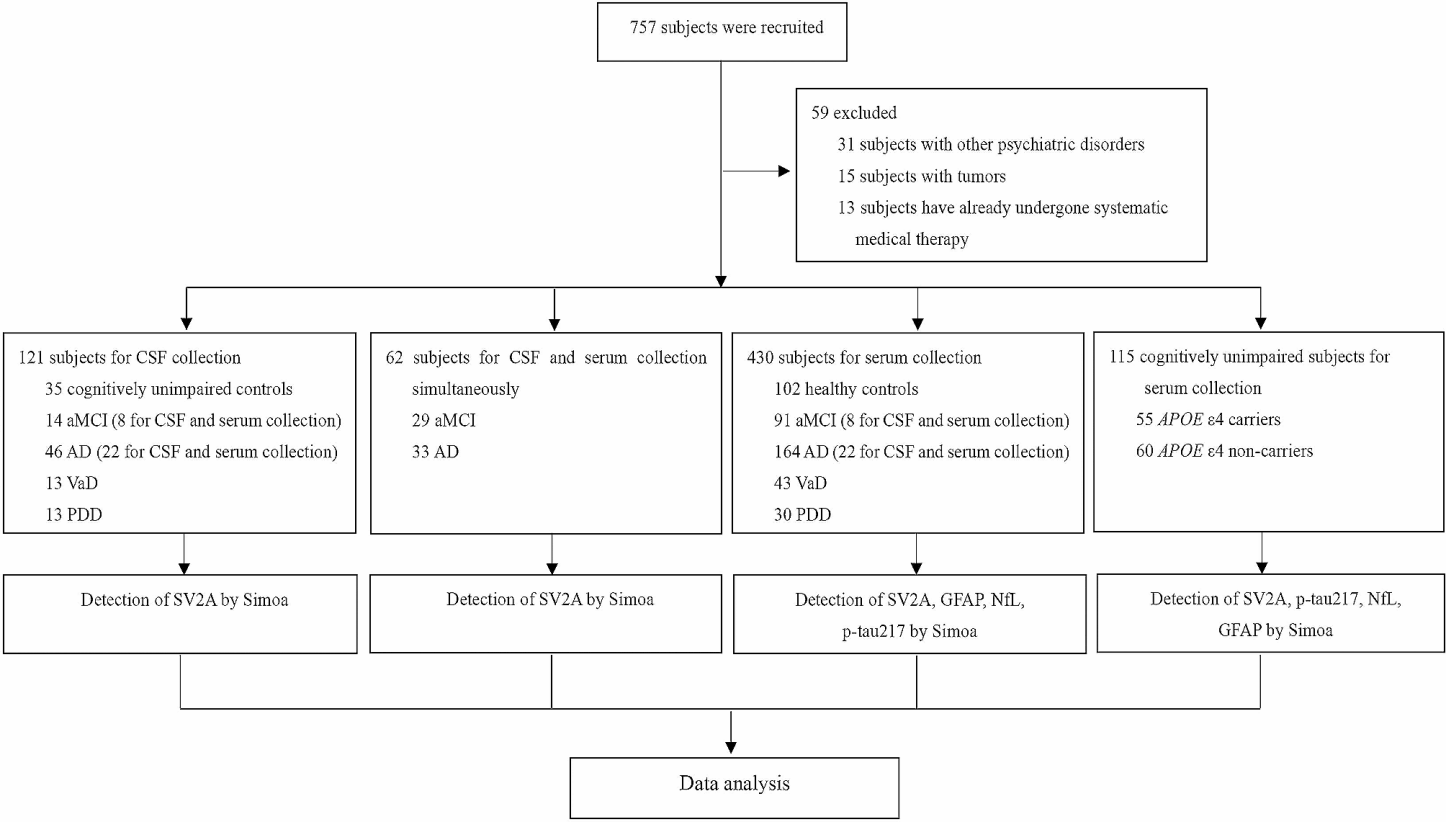
Flowchart for subject enrolment and exclusion Abbreviations: AD, Alzheimer’s disease; aMCI, amnestic mild cognitive impairment; CSF, cerebrospinal fluid; GFAP, glial fibrillary acidic protein; NfL, neurofilament light; PDD, Parkinson’s disease dementia; Simoa, single molecule array; SV2A, synaptic vesicle glycoprotein 2 A; VaD, vascular dementia.

In total, 59 individuals were excluded, and 698 were finally included in the study. The sera of 430 participants comprising the healthy control (*n* = 102), aMCI (*n* = 91), AD (*n* = 164), VaD (*n* = 43), and PDD (*n* = 30) groups were investigated. The CSF samples of 121 patients comprising the normal cognition group (*n* = 35), aMCI (*n* = 14), AD (*n* = 46), VaD (*n* = 13), and PDD (*n* = 13) were also analyzed. Of these, a total of 8 patients with aMCI and 22 with AD had their CSF and venous blood samples collected simultaneously and then tested for SV2A, and we collected CSF and serum samples from an additional 29 aMCI and 33 AD patients simultaneously for correlation analysis between CSF SV2A and serum SV2A. In addition, the sera of 115 participants with unimpaired cognition, including apolipoprotein E *(APOE)* ε4 carriers (*n* = 55) and non-carriers (*n* = 60), were examined. The subject inclusion and exclusion process is shown in Fig. 1. No statistically significant difference in age was found between the dementia groups and the control group, and between *APOE* ε4 carriers and *APOE* ε4 non-carriers. The sex ratio of each dementia group matched that of the control group, except for the VaD group that had undergone blood testing. Clinical and demographic features of the diagnostic cohorts are shown in Table 1.

**Table 1:** Clinical and demographic features of the diagnostic cohorts

	Con	aMCI	AD	VaD	PDD
Serum					
** No.**	102	91	164	43	30
** Sex, female/male**	57/45	51/40	87/77	13/30**	15/15
** Age, years (SD)**	65.45 ± 7.88	66.66 ± 7.09	67.20 ± 8.63	68.42 ± 8.94	64.37 ± 8.43
** Education level, years (SD)**	NA	12.94 ± 3.58	11.11 ± 3.83	10.95 ± 4.66	11.17 ± 3.43
** MMSE score, mean (SD)**	NA	27.96 ± 1.04	17.14 ± 6.34	20.42 ± 5.04	23.20 ± 2.76
** MOCA score, mean (SD)**	NA	24.02 ± 2.10	12.21 ± 5.99	15.74 ± 5.35	19.97 ± 4.98
** Serum SV2A, mean (pg/mL) (SD)**	5297.76 ± 3734.08	2367.11 ± 1601.60**	1327.56 ± 1290.37**	4497.22 ± 3059.41^††^	3974.93 ± 2952.57^††^
** Serum NfL, mean (pg/mL) (SD)**	13.77 ± 9.89	16.30 ± 9.91*	20.33 ± 14.78**	38.54 ± 39.64**^††^	12.22 ± 6.73^††^
** Serum GFAP, mean (pg/mL) (SD)**	8.28 ± 7.55	15.22 ± 8.45**	20.78 ± 10.54**	15.12 ± 10.27**^††^	12.99 ± 8.48**^††^
** Serum p-tau217, mean (pg/mL) (SD)**	1.68 ± 1.37	3.67 ± 1.69**	4.38 ± 2.50**	3.53 ± 3.04**^†^	4.44 ± 2.60**
**CSF**					
** No.**	35	14	46	13	13
** Sex, female/male**	18/17	8/6	31/15	5/8	4/9
** Age, years (SD)**	62.23 ± 8.43	64.86 ± 8.52	61.80 ± 7.37	67 ± 10.81	64.92 ± 8.80
** Education level, years (SD)**	NA	12.79 ± 2.21	10.22 ± 4.35	10.08 ± 4.34	11.92 ± 3.83
** MMSE score, mean (SD)**	NA	27.96 ± 1.04	17.14 ± 6.34	20.42 ± 5.04	23.20 ± 2.76
** MOCA score, mean (SD)**	NA	24.02 ± 2.10	12.21 ± 5.99	15.74 ± 5.35	19.97 ± 4.98
** CSF SV2A, mean (pg/mL) (SD)**	7223.10 ± 2150.76	5279.63 ± 876.46**	3109.15 ± 1819.14**	6288.36 ± 1818.16^††^	6395.49 ± 1472.95^††^

Note: The normality of the distribution of the variables was assessed by the Shapiro–Wilk test. Continuous variables were compared between two independent samples using the t-test or the Mann–Whitney U test. Chi-square test was used to assess sex. Logistic regression models were employed to compare continuous variables between different groups before and after adjusting for covariates such as age and sex. *p < 0.05 and **p < 0.01, compared with control; ^†^*p* < 0.05 and ^††^*p* < 0.01, compared with AD.

Abbreviations: AD, Alzheimer’s disease; aMCI, amnestic mild cognitive impairment; Con, control subjects; GFAP, glial fibrillary acidic protein; MMSE, Mini-Mental State Examination; MoCA, Montreal Cognitive Assessment; NA, not available; NfL, neurofilament light; p-tau217, phosphorylated tau; PDD, Parkinson’s disease dementia; SD, standard deviation; VaD, vascular dementia. SV2A, synaptic vesicle glycoprotein 2 A

### CSF and serum collection

CSF samples were immediately collected according to international guidelines [25]. Briefly, the participants were placed in the left lateral position for lumbar puncture. The L3–L5 intervertebral disc spaces were chosen as the puncture site. CSF samples were collected with 20-gauge atraumatic needles and centrifugated at 2000 × g for 10 min at room temperature.

Blood samples were drawn by venipuncture in the morning after a 12-h fast and were kept in serum tubes with a clot activator. Sera were extracted from the blood samples by centrifugation at 2000 × g for 10 min. CSF and serum samples were then stored in polypropylene tubes at − 80℃ until the next test, avoiding repeated freezing and thawing.

### Genetic analyses

Genomic DNA was extracted from the peripheral blood samples using a salting-out procedure [26]. *APOE* genotypes were determined by Sanger sequencing, and the primers used were as follows: forward—AGACGCGGGCACGGCTGTCCAAGGA; reverse—AGACGCGGGCACGGCTGTCCAAGGA [27].

### Simoa assay for CSF and serum analysis

For the novel SV2A Simoa assay, a polyclonal antibody specifically recognizing SV2A was used (CSB-PA022978LA01HU, CUSABIO, Wuhan, China) as capture antibody, with identifying sequence of 36-149AA. Another rabbit Anti-SV2A antibody (bs-2407R, BIOSS, Beijing, China) was used as a detection antibody with a recognition site of 451–550. CSF and serum samples were analyzed on the AST-Sc-Lite, a fully-auto single-molecule detection machine (Suzhou AstraBio Technology Co., Ltd., China), according to the manufacturer’s instructions. GFAP and NfL were detected as described in the previously published study [28]. Briefly, the working steps were as follows: (I) load 25 µL of the sample into an incubation tube and add reagent 1 (mainly comprised of 0.1 mg/mL magnetic beads coated with capture antibodies and protecting reagents), followed by a quick mixing by the machine. (II) After 6 min of incubation, reagent 2 (mainly comprised of the detection antibodies labeled with single-molecule imagine fluorophores supplied by AstraBio) was added, mixed, and incubated for 4 min at 40 °C. (III) The magnetic beads in the mixtures were absorbed onto the surface of the channel in the flow cell by a permanent magnet. Unlabeled fluorophores were removed by gentle washing flow of the wash buffer, and fluorescent images were then taken with an integrated fluorescent microscope. (IV) The single-molecule signals were analyzed by the machine, and protein concentrations were calculated using a build standard curve prepared in advance. The standard curves for each biomarker were shown in Additional file 1: Fig. S1. The new SV2A simoa assay as well as the GFAP, NfL and p-tau217 simoa assays demonstrated excellent detection performance, and more information was presented in Additional file 1.

### Stereotaxic injection of adeno-associated virus (AAV)

As for the construction of AAV9-SV2Aoe, the vector HBAAV9-m-3xflag-T2A-mcherry (Hanbio Biotechnology, Shanghai, China) was used. 9-month-old APP/PS1 mice prepared for the stereotaxic injection were deeply anesthetized with an intraperitoneal injection of sodium pentobarbital (50 mg/kg body weight) and secured in a stereotaxic apparatus. The AAV9 were bilaterally injected into the dorsal hippocampal area at a rate of 0.2 µL/min, and the total volumes of AAV-SV2Aoe (1.3 × 10^12^ vg/mL) and AAV-Con (1.9 × 10^12^ vg/mL) injected into the unilateral hippocampal region were 1.5 µL and 1.0 µL, respectively. The needle was retracted after 10 min, and the wound was sutured after another 10 min.

### Histology and immunostaining

4% paraformaldehyde-fixed mice brain tissues were dehydrated in 30% sucrose and embedded in the optimal cutting temperature compound for frozen sectioning. Antigen retrieval was performed by using citric acid (pH 6.0) antigen repair solution in a sub-boiling temperature. Then sections were immersed in 3% hydrogenperoxide to block the endogenous peroxidase activity followed by incubation in 3% bovine serum albumin solution to block non-specific binding. The slides were then incubated overnight at 4 °C in a wet box with rabbit anti-6E10 (Cat. No. 803,015, BioLegend, USA, 1:1000). HRP-labelled goat anti-rabbit IgG secondary antibodies (Cat. No. GB23303, Servicebio, China, 1:500) were applied for 50 min at room temperature in dark condition, and antigen was visualized using tyramide signal amplification kits (Cat. No. G1226, Servicebio) according to the manufacturer’s protocol. Sections were imaged using a slide scanner (PANNORAMIC, 3D HISTECH, Hungary). Images were then analyzed by Image J software, and the background was subtracted by the software for fluorescence images before quantification. The number and the size of Aβ plaques, and the fluorescence intensity of 6E10 in three brain sections containing the hippocampus and cortex of each mouse were measured.

### ELISA

The blood of mice was collected into polypropylene tubes by the eyeball blood sampling method, left at room temperature for 2 h, then centrifuged at 3000 rpm/min for 20 min, and the supernatant was aspirated into new polypropylene tubes and stored at -80 °C to avoid repeated freezing and thawing. Levels of Aβ40 (Cat. No. EM0863, FineTest, Wuhan, China) and Aβ42 (Cat. No. EM0864, FineTest, Wuhan, China) were measured by ELISA following the manufacturer’s instructions.

### Statistical analysis

The normality of the distribution of the variables was assessed using the Shapiro–Wilk test. Continuous variables were compared between two independent samples using the t-test or the Mann–Whitney U test. Categorical data were analyzed using the chi-square test. Diagnostic accuracy was evaluated using receiver operating characteristic (ROC) curve analysis, and representative optimal sensitivity and specificity were used to evaluate the performance of the models. Cutoff levels were determined by maximizing the Youden index (sensitivity + specifcity − 1). Logistic regression was used to evaluate the predictive models, and ROC curves were constructed from the logistic scores. Logistic regression models were employed to compare continuous variables between different groups before and after adjusting for covariates such as age and sex. Partial correlation analyses or Spearman’s rank correlation analyses were performed to assess the correlations between biomarkers, demographic characteristics, and clinical data. The statistical significance of the difference in the areas under the curve (AUCs) between two different models was analyzed using Delong’s test. The parallel test and serial test were used to calculate the sensitivity and specificity of the combined diagnostic models, respectively. All tests were two-tailed, and *p* < 0.05 was considered statistically significant. All analyses were performed using IBM SPSS Statistics version 24 (IBM Corp., Armonk, NY, USA) and MedCalc version 20.0.22. Data were visualized using Prism 9 (GraphPad, San Diego, CA, USA).

## Results

### SV2A levels decreased significantly in both CSF and serum with AD progression

**Fig. 2:**
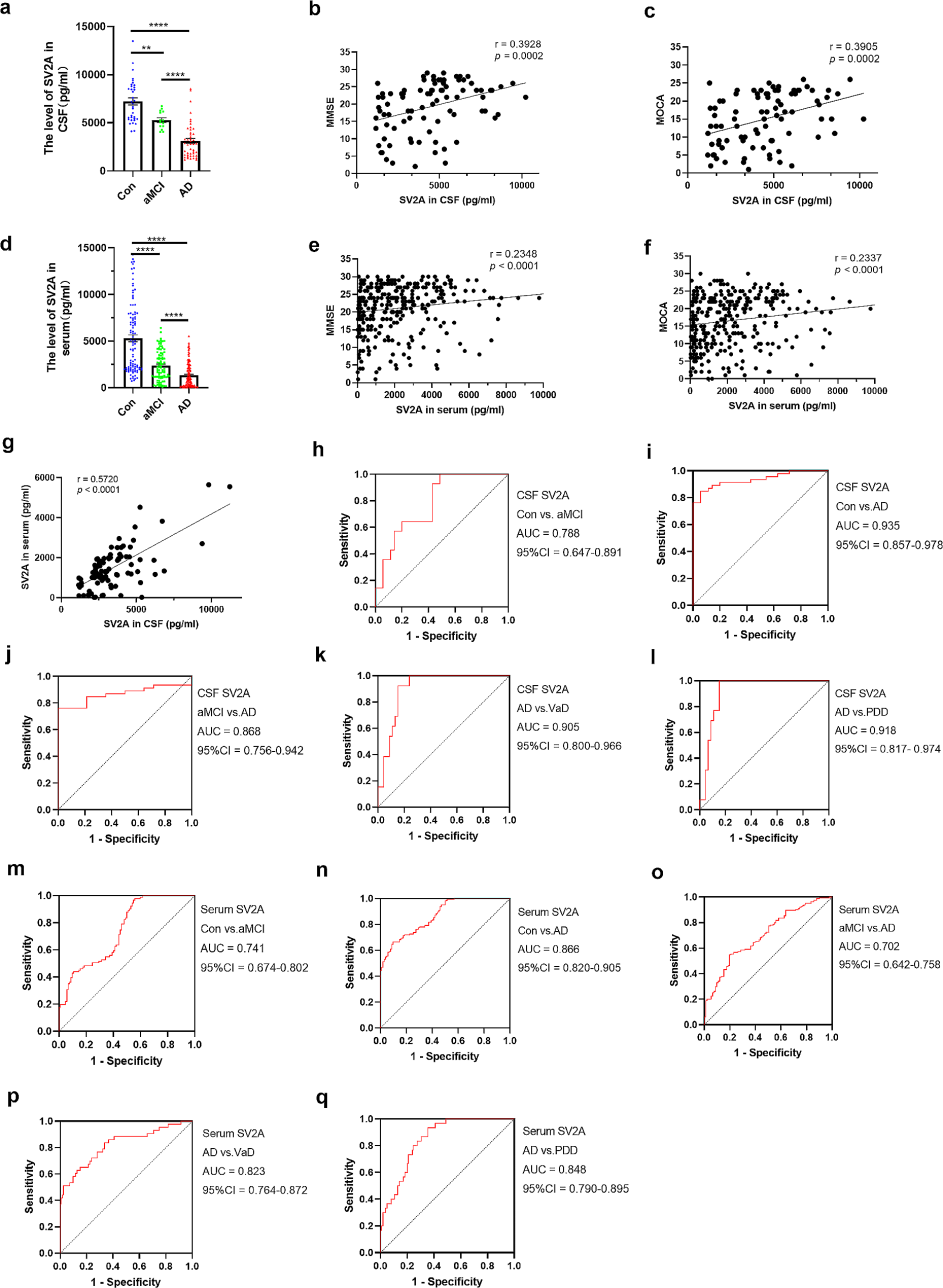
SV2A levels in CSF and serum at different stages of AD and the early diagnostic and differential diagnostic efficacy of them for AD. **a**. CSF SV2A levels at different stages of AD (Con = 35, aMCI = 14, AD = 46). **b.** Correlation of CSF SV2A with MMSE scores. **c.** Correlation of CSF SV2A with MOCA scores. **d**. Serum SV2A at different stages of AD (Con = 102, aMCI = 91, AD = 164). (**e**) Correlation of serum SV2A level with MMSE scores. (**f**) Correlation of serum SV2A level with MOCA scores. (**g**) Correlation analysis of CSF SV2A and serum SV2A (aMCI, *n* = 37; AD, *n* = 55). **h–l.** Diagnostic efficacy of CSF SV2A for Con vs. aMCI, Con vs. AD, aMCI vs. AD, VaD vs. AD, and PDD vs. AD. **m–q.** Diagnostic efficacy of serum SV2A for Con vs. aMCI, Con vs. AD, aMCI vs. AD, VaD vs. AD, and PDD vs. AD. CSF SV2A and serum SV2A were presented as means ± SEM. The significance of the between-group differences was determined using the Mann–Whitney U test. Partial correlation analyses were performed to assess the correlations between biomarkers and cognitive scores by controlling for confounders age and sex. *p < 0.05, **p < 0.01, ***p ≤ 0.001, ****p ≤ 0.0001. Abbreviations: 95% CI, 95% confidence interval; AD, Alzheimer’s disease; AUC, area under the curve; Con, control; aMCI, amnestic mild cognitive impairment; CSF, cerebrospinal fluid; PDD, Parkinson’s disease dementia; SV2A, synaptic vesicle glycoprotein 2 A; vs., versus

In this study, CSF SV2A levels in 14 patients with aMCI, 46 patients with AD, and 35 age-matched controls were first examined by the Simoa method. The results showed that SV2A levels in the CSF gradually decreased with the severity of dementia. Compared with the control group, mean levels of CSF SV2A in the aMCI and AD groups were significantly reduced by approximately 26.91% (*p* = 0.0013) and 56.96% (*p* < 0.0001), respectively. Compared with the mean level in the aMCI group, the mean level of CSF SV2A in the AD group was reduced by approximately 41.11% (*p* < 0.0001) (Fig. 2a). To reveal the relationship between CSF SV2A and cognitive ability in the AD group, a correlation analysis was also performed between CSF SV2A and cognitive scores, which showed a significant positive correlation between CSF SV2A levels and MMSE (*r* = 0.3928, *p* = 0.0002) and MOCA (*r* = 0.3905, *p* = 0.0002) (Fig. 2b-c).

To further explore SV2A alterations in the blood, serum samples from 91 patients with aMCI, 164 patients with AD, and 102 age-matched controls were subsequently tested for SV2A using the Simoa method. Consistent with the trend in CSF, serum SV2A levels also progressively decreased with the progression of dementia, as evidenced by a significant reduction in the mean serum SV2A levels of approximately 55.32% (*p* < 0.0001) and 74.94% (*p* < 0.0001) in aMCI and AD, respectively, relative to controls, and a significant reduction of approximately 43.92% (*p* < 0.0001) in AD relative to aMCI (Fig. 2d). In the correlation analyses with cognitive scores, serum SV2A, which was similar to CSF SV2A, significantly and positively correlated with both MMSE (*r* = 0.2348, *p* < 0.0001) and MOCA (*r* = 0.2337, *p* < 0.0001) (Fig. 2e-f).

Based on the above results, SV2A showed high concordance between blood and CSF; therefore, a correlation analysis of 92 patients (including 37 patients with aMCI and 55 with AD) was performed in which both CSF and serum were tested. The results showed a significant positive correlation between serum SV2A and CSF SV2A (*r* = 0.5720, *p* < 0.0001) (Fig. 2g). The detailed data were presented in Table 1 and all these data were adjusted for age and sex.

### CSF and serum SV2A had significant early diagnostic and differential diagnostic efficacy for AD

Since SV2A was closely associated with cognitive impairment in patients with AD, the early diagnostic and differential diagnostic ability of SV2A for AD was also analyzed. Diagnostic accuracy was evaluated using ROC curve analysis, and the Youden index was calculated to determine the best cutoff regarding sensitivity and specificity. Initially, CSF SV2A demonstrated significant diagnostic ability for aMCI (AUC = 78.8%, 95% CI = 0.647–0.891, sensitivity = 100.00%) (Fig. 2h) and differential diagnosis of aMCI from AD (AUC = 86.8%, 95% CI = 0.756–0.942) (Fig. 2j). CSF SV2A also showed high diagnostic performance for AD dementia (AUC = 93.5%, 95% CI = 0.857–0.978, sensitivity = 84.78%, specificity = 94.26%) (Fig. 2i). Although the mean level of CSF SV2A was lower in the VaD and PDD groups than in the cognitively unimpaired control group, the difference was not statistically significant (Table 1). Then, the CSF SV2A levels in the AD, VaD, and PDD groups were compared, revealing that the average CSF SV2A level was significantly lower in the AD group than in the other two groups, which demonstrated high diagnostic efficacy in the differential diagnosis of AD from VaD (AUC = 90.5%, 95% CI = 0.800–0.966, sensitivity = 84.78%, specificity = 92.31%) (Fig. 2k) and PDD (AUC = 91.8%, 95% CI = 0.817–0.974, sensitivity = 84.78%, specificity = 100.00%) (Fig. 2l).

Considering the invasive nature of CSF sampling, we next explored whether serum SV2A could be used as a screening indicator for early-stage AD. Surprisingly, like CSF SV2A, serum SV2A also demonstrated statistical significance in the diagnosis of aMCI (AUC = 74.1%, 95% CI = 0.674–0.802, sensitivity = 97.80%) (Fig. 2m) and differential diagnosis of aMCI from AD (AUC = 70.2%, 95% CI = 0.642–0.758) (Fig. 2o). Serum SV2A also demonstrated the same diagnostic efficacy as CSF SV2A in the diagnosis of AD (AUC = 86.6%, 95% CI = 0.820–0.905) (Fig. 2n). Compared with the significant decrease in AD, although the mean level of serum SV2A decreased in VaD and PDD relative to cognitively normal controls, it was not statistically significant. However, the mean level of serum SV2A, consistent with the trend in the CSF, was significantly lower in the AD group than in the VaD and PDD groups (Table 1), with good diagnostic efficacy in identifying AD from VaD (AUC = 82.3%, 95% CI = 0.764–0.872) (Fig. 2p) and PDD (AUC = 84.8%, 95% CI = 0.790–0.895) (Fig. 2q). All data are shown in Tables 1 and 2.

**Table 2:** Efficacy of SV2A for the early diagnosis and differential diagnosis of AD

	CSF SV2A						Serum SV2A				
	AUC (95% CI)	*p*-value	Cutoff (pg/mL)	SEN (%)	SPE (%)		AUC (95% CI)	*p*-value	Cutoff (pg/mL)	SEN (%)	SPE (%)
**Con vs. aMCI**	0.788 (0.647–0.891)	**< 0.0001**	≤ 6726.35	100.00	51.43		0.741 (0.674–0.802)	**< 0.0001**	≤ 5050.24	97.80	44.12
**Con vs. AD**	0.935 (0.857–0.978)	**< 0.0001**	≤ 4526.64	84.78	94.26		0.866 (0.820–0.905)	**< 0.0001**	≤ 1674.02	66.46	90.20
**aMCI vs. AD**	0.868 (0.756–0.942)	**< 0.0001**	≤ 3960.97	76.09	100		0.702 (0.642–0.758)	**< 0.0001**	≤ 1009.39	54.88	80.22
**VaD vs. AD**	0.905 (0.800–0.966)	**< 0.0001**	≤ 4526.64	84.78	92.31		0.823 (0.764–0.872)	**< 0.0001**	≤ 1674.02	66.46	83.72
**PDD vs. AD**	0.918 (0.817–0.974)	**< 0.0001**	≤ 4526.64	84.78	100.00		0.848 (0.790–0.895)	**< 0.0001**	≤ 1581.51	64.63	93.33

Note: Diagnostic accuracy was evaluated using the receiver operating characteristic (ROC) curve analysis. The Youden index was calculated to determine the best cutoff regarding sensitivity and specificity. Abbreviations: 95% CI, 95% confidence interval; AD, Alzheimer’s disease; aMCI, amnestic mild cognitive impairment; AUC, area under the curve; Con, control; CSF, cerebrospinal fluid; PDD, Parkinson’s disease dementia; SEN, sensitivity; SPE, specificity; SV2A, synaptic vesicle glycoprotein 2 A; VaD, vascular dementia

### SV2A demonstrated high positivity rates in patients with aMCI who were negative for other biomarkers

The high diagnostic sensitivity (97.80%) in aMCI suggested that serum SV2A was valuable for the early screening of aMCI. To further support this point, we simultaneously tested other AD core biomarkers in the sera of the above diagnostic groups, including NfL, GFAP, and p-tau217 (Table 1). Then, we speculated whether serum SV2A could correct aMCI cases that were negative for other biomarkers. We counted the number of other biomarker-negative patients in the aMCI group and of which the number of SV2A-positive patients, respectively. Briefly, patients with serum NfL concentrations below its cutoff value (≤ 9.68 pg/mL in the aMCI group) (Table 3) were considered as serum NfL test-negative aMCI, whereas patients with serum SV2A concentrations below its cutoff value (≤ 5050.24 pg/mL in the aMCI group) (Table 2) were regarded as serum SV2A-positive aMCI. Statistical results showed that serum SV2A demonstrated an extremely high positivity rate of 100.00% in the NfL-negative cases of aMCI. By the same method, patients with concentrations below the cutoff value (≤ 7.67 pg/mL for GFAP and ≤ 3.24 pg/mL for p-tau217) (Table 3) were regarded as GFAP- and p-tau217 test-negative aMCI, respectively, and we found that serum SV2A was positive in 92.86% and 97.06% of GFAP and p-tau217-negative aMCI cases, respectively. In addition, we also calculated the positivity rates of serum SV2A in AD cases that were negative for other biomarkers by the same method, however, the rate was lower than that in the aMCI cases. All data on the positivity rate was presented in Table 4.

**Table 3:** Efficiency of serum SV2A in combination with other biomarkers in the diagnosis of aMCI or AD, and the differential diagnosis of AD from other dementias

	Con vs. aMCI	Con vs. AD	aMCI vs. AD	AD vs. VaD	AD vs. PDD
**NfL**					
** AUC (95%CI)**	0.590 (0.517–0.660)	0.645 (0.584–0.702)	0.564 (0.501–0.626)	0.649 (0.579–0.714)	0.679 (0.608–0.744)
** Cutoff (pg/mL)**	> 9.68	> 15.53	> 17.66	≤ 18.20	> 19.15
** SEN (%)**	73.63	56.10	47.56	67.44	90.00
** SPE (%)**	44.12	68.63	64.84	54.88	43.29
**GFAP**					
** AUC (95%CI)**	0.777 (0.712–0.834)	0.859 (0.811–0.898)	0.663 (0.602–0.721)	0.663 (0.595–0.727)	0.725 (0.657–0.787)
** Cutoff (pg/mL)**	> 7.67	> 12.93	> 20.88	> 19.62	> 11.07
** SEN (%)**	84.62	75.61	45.12	79.07	56.67
** SPE (%)**	63.73	83.33	83.52	50.61	81.10
**p-tau217**					
** AUC (95%CI)**	0.832 (0.771–0.882)	0.862 (0.814–0.901)	0.561 (0.497–0.622)	0.631 (0.561–0.697)	0.501 (0.428–0.573)
** Cutoff (pg/mL)**	> 3.24	> 3.09	> 6.24	> 2.85	NA
** SEN (%)**	62.64	67.68	18.29	60.47	NA
** SPE (%)**	89.22	87.25	95.60	70.73	NA
**SV2A + NfL**					
** AUC (95%CI)**	0.771 (0.705–0.828)	0.883 (0.838–0.919)	0.734 (0.676–0.787)	0.856 (0.801–0.901)	0.868 (0.812–0.912)
** Cutoff (pg/mL)**	NA	NA	NA	NA	NA
** SEN (%)**	100.0*	89.0*	83.5*	80.5*	83.5*
** SPE (%)**	71.6^†^	98.0^†^	97.8^†^	97.7^†^	96.7^†^
**SV2A + GFAP**					
** AUC (95%CI)**	0.865 (0.809–0.910)	0.937 (0.900–0.963)	0.764 (0.707–0.814)	0.836 (0.779–0.884)	0.881 (0.827–0.923)
** Cutoff (pg/mL)**	NA	NA	NA	NA	NA
** SEN (%)**	98.9*	93.3*	78.7*	86.6*	93.3*
** SPE (%)**	81.4^†^	98.0^†^	97.8^†^	93.0^†^	100.0^†^
**SV2A + p-tau217**					
** AUC (95%CI)**	0.891 (0.838–0.931)	0.942 (0.906–0.967)	0.727 (0.667–0.780)	0.819 (0.760–0.869)	0.855 (0.797–0.901)
** Cutoff (pg/mL)**	NA	NA	NA	NA	NA
** SEN (%)**	98.9*	93.3*	65.2*	93.9*	88.4*
** SPE (%)**	95.1^†^	100.0^†^	100.0^†^	93.0^†^	93.3^†^
**SV2A + GFAP + NfL + p-tau217**					
** AUC (95%CI)**	0.918 (0.870–0.952)	0.962 (0.931–0.982)	0.790 (0.735–0.838)	0.864 (0.810–0.908)	0.896 (0.844–0.935)
** Cutoff (pg/mL)**	NA	NA	NA	NA	NA
** SEN (%)**	100.0*	100.0*	92.1*	98.2*	97.6*
** SPE (%)**	98.0^†^	100.0^†^	100.0^†^	100.0^†^	100.0^†^

Note: Logistic regression was used to evaluate predictive models and receiver operating characteristic (ROC) curves constructed from the logistic scores. The Youden index was calculated to determine the best cutoff regarding sensitivity and specificity for the single indicator. The *parallel test and ^†^serial test was used to calculate the sensitivity and specificity of the combined diagnostic models, respectively. Abbreviations: 95% CI, 95% confidence interval; AD, Alzheimer’s disease; aMCI, amnestic mild cognitive impairment; AUC, area under the curve; GFAP, glial fibrillary acidic protein; NfL, neurofilament light; NA, not available; PDD, Parkinson’s disease dementia; p-tau217, phosphorylated tau; SEN, sensitivity; SPE, specificity; SV2A, synaptic vesicle glycoprotein 2 A; VaD, vascular dementia; vs., versus

**Table 4:** Positive rates of serum SV2A in cases negative for other biomarkers

	aMCI	AD
**SV2A (+) / NfL (−)**	100.00% (24/24)	75% (54/72)
**SV2A (+) / GFAP (−)**	92.86% (13/14)	72.50% (29/40)
**SV2A (+) / p-tau217 (−)**	97.06% (33/34)	79.25% (42/53)

Abbreviations: AD, Alzheimer’s disease; aMCI, amnestic mild cognitive impairment; GFAP(−), serum GFAP-negative patients (≤ 7.67 pg/mL in aMCI, ≤ 12.93 pg/mL in AD); GFAP, glial fibrillary acidic protein; NfL(−), serum NfL-negative patients (≤ 9.68 pg/mL in aMCI, ≤ 15.53 pg/mL in AD); NfL, neurofilament light; p-tau217(−), serum p-tau217-negative patients (≤ 3.24 pg/mL in aMCI, ≤ 3.09 pg/mL in AD); p-tau217, phosphorylated tau; SV2A (+), serum SV2A-positive patients (≤ 5050.24 pg/mL in aMCI, ≤ 1674.02 pg/mL in AD); SV2A, synaptic vesicle glycoprotein 2 A

### Serum SV2A combined with other biomarkers significantly improved the early diagnosis efficiency of AD

**Fig. 3:**
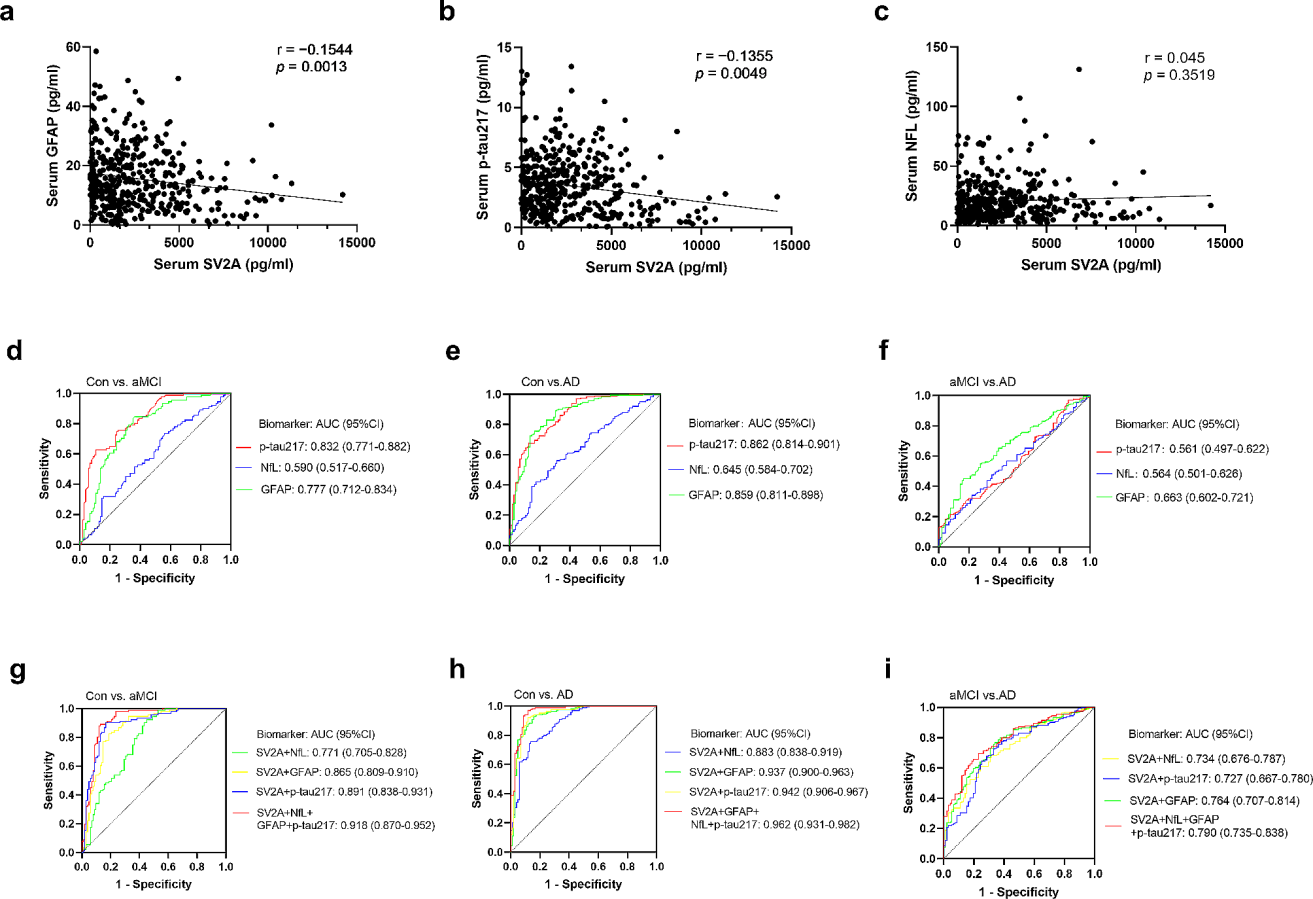
Correlation analysis of serum SV2A with other biomarkers, and the diagnostic efficiency of serum SV2A combined with these biomarkers in aMCI and AD. Relationship of serum SV2A with (**a**) serum GFAP, (**b**) serum p-tau217, and (**c**) serum NfL. **d.** Diagnostic efficiency of serum NfL, GFAP, and p-tau217 in Con vs. aMCI. **e.** Diagnostic efficiency of serum NfL, GFAP, and p-tau217 in Con vs. AD. **f.** Diagnostic efficiency of serum NfL, GFAP, and p-tau217 in aMCI vs. AD. **g.** Diagnostic efficiency of serum SV2A + NfL, SV2A + GFAP, SV2A + p-tau217, and SV2A + NfL + GFAP + p-tau217 in Con vs. aMCI. **h.** Diagnostic efficiency of serum SV2A + NfL, SV2A + GFAP, SV2A + p-tau217, and SV2A + NfL + GFAP + p-tau217 in Con vs. AD. **i.** Diagnostic efficiency of serum SV2A + NfL, SV2A + GFAP, SV2A + p-tau217, and SV2A + NfL + GFAP + p-tau217 in aMCI vs. AD. Correlation analysis was performed using Spearman’s rank correlation coefficient. Abbreviations: AD, Alzheimer’s disease; aMCI, amnestic mild cognitive impairment; AUC, area under the curve; Con, healthy control; GFAP, glial fibrillary acidic protein; NfL, neurofilament light; p-tau217, phosphorylated tau; SV2A, synaptic vesicle glycoprotein 2 A; vs., versus

The above results indicated that SV2A demonstrated perfect complementarity with other biomarkers in the early diagnosis of AD, we further explored whether combining serum SV2A with other biomarkers could improve the diagnosis efficacy for aMCI. First, the correlation analysis showed that serum SV2A significantly negatively correlated with serum GFAP (*r* = − 0.1544, *p* = 0.0013) and serum p-tau217 (*r* = − 0.1355, *p* = 0.0049), respectively (Fig. 3a-b). Although serum SV2A did not demonstrate a significant correlation with NfL (Fig. 3c), the diagnostic models combining serum SV2A with NfL (GFAP or p-tau217) had significantly higher diagnostic AUCs than their corresponding single-indicator models (*p* < 0.01) (Fig. 3d-g). When serum SV2A was combined with the above three biomarkers, the AUC for aMCI was further improved to 91.8%, which was significantly higher than that of NfL, GFAP, or p-tau217 alone, as well as the diagnostic model combining the three biomarkers (*p* < 0.01) (Additional file 1: Table S1). The high sensitivity (97.80%) suggests that serum SV2A could be an excellent early screening biomarker for aMCI, whereas the low specificity (44.12%) will somewhat reduce its efficacy. Therefore, we tried to find indicators that could make up for this shortcoming. Compared with other indicators tested, serum p-tau217 had higher specificity (89.22%) for aMCI, and the diagnostic model combining serum SV2A and p-tau217 significantly increased the diagnostic specificity of aMCI to 95.1% by the serial test, which was further improved to 98% when serum SV2A was combined with the other three indicators (Table 3).

In addition to enhancing the diagnostic efficacy of aMCI in the cognitively normal populations, the combinations of serum SV2A with other biomarkers significantly improved the ability to identify aMCI from AD. Initially, among biomarkers tested, serum SV2A demonstrated the highest diagnostic AUC for identifying aMCI and AD, which was significantly higher than that of NfL (*p* = 0.0159) and p-tau217 (*p* = 0.0096) (Additional file 1: Table S2). After combining with serum SV2A, the AUCs of NfL, GFAP, and p-tau217 for the differential diagnosis between aMCI and AD significantly improved to 73.4%, 76.4%, and 72.7%, respectively (*p* < 0.01) (Fig. 3i). Although the combination of serum SV2A with other biomarkers could improve the sensitivity of distinguishing aMCI from AD to some extent, as a differential diagnostic marker, its specificity was more deserving of attention. By the serial test, the specificity of SV2A in identifying aMCI from AD was significantly improved when combined with other markers, especially with p-tau217, which increased the specificity from 80.22 to 100.0% (Table 3).

In the diagnosis of AD, the AUCs of the diagnostic models combining serum NfL, GFAP, and p-tau217 with serum SV2A were improved to 88.3%, 93.7%, and 94.2%, respectively, which were significantly higher than the AUCs of these biomarkers when used alone (*p* < 0.01). Excitingly, the combination of these four biomarkers resulted in a significant increase in the diagnostic AUC for AD to 96.2% (*p* < 0.01) (Fig. 3h and Additional file 1: Table S3). The specificity of 90.2% made serum SV2A an excellent diagnostic indicator for AD, and by the parallel test, its sensitivity significantly increased from 66.46 to 89.0%, 93.0%, and 93.0% after combining with NfL, GFAP, and p-tau217, respectively. The diagnostic model combining the above four biomarkers achieved a sensitivity of 100.0% by the parallel test (Table 3).

### Serum SV2A combined with other biomarkers significantly improved the differential diagnosis efficiency of AD from non-AD dementia

**Fig. 4:**
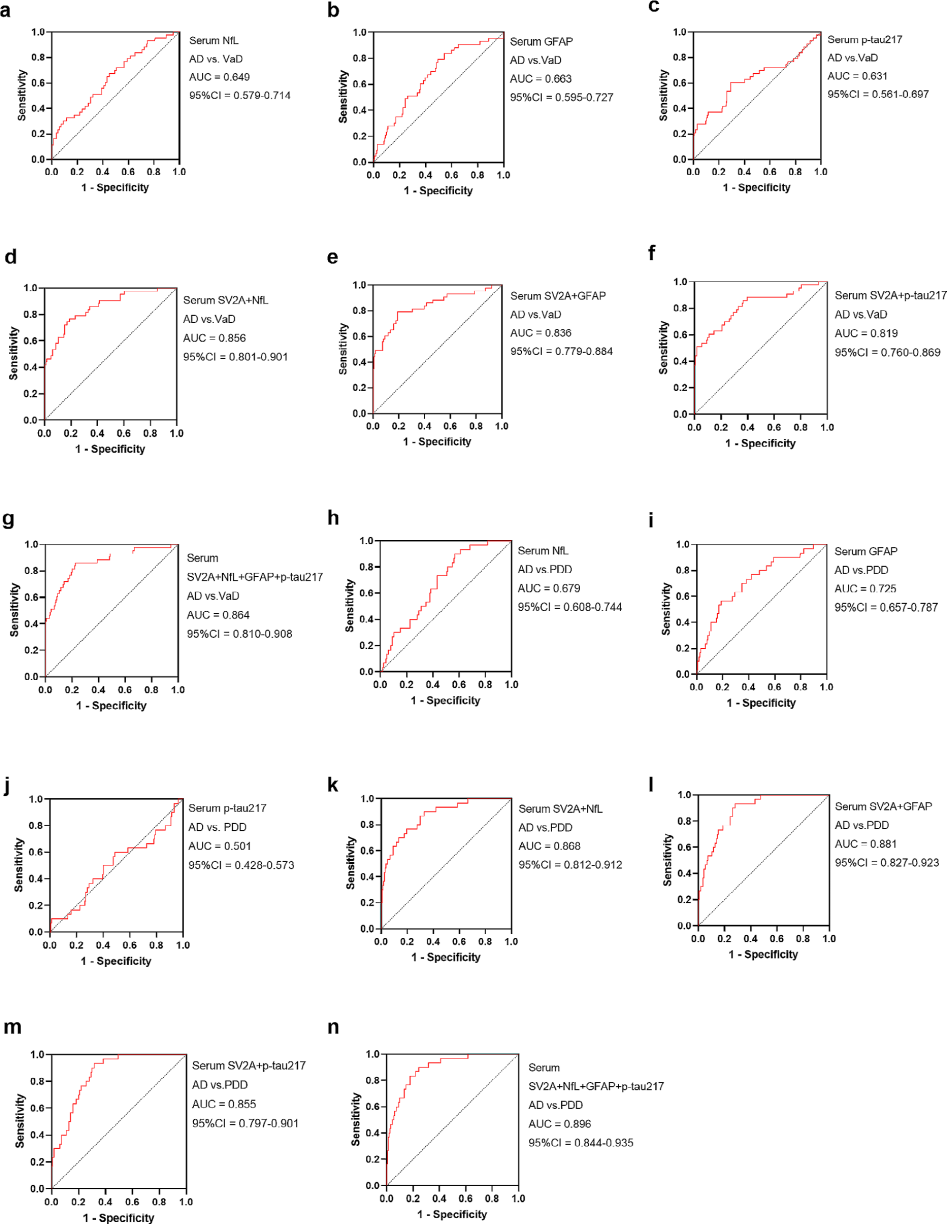
Differential diagnostic efficiency of serum SV2A combined with other biomarkers in AD vs. VaD and AD vs. PDD. **a–c**. Differential diagnostic efficiency of serum NfL, GFAP, and p-tau217 in AD vs. VaD. **d–g.** Differential diagnostic efficiency of serum SV2A + NfL, SV2A + GFAP, SV2A + p-tau217, and SV2A + NfL + GFAP + p-tau217 in AD vs. VaD. **h–j.** Differential diagnostic efficiency of serum NfL, GFAP, and p-tau217 in AD vs. PDD. **k–n.** Differential diagnostic efficiency of serum SV2A + NfL, SV2A + GFAP, SV2A + p-tau217, and SV2A + NfL + GFAP + p-tau217 in AD vs. PDD. Abbreviations: 95% CI, 95% confidence interval; AD, Alzheimer’s disease; AUC, area under the curve; PDD, Parkinson’s disease dementia; SV2A, synaptic vesicle glycoprotein 2 A; VaD, vascular dementia; vs., versus

As mentioned previously, serum SV2A demonstrated significant efficacy in the differential diagnosis of AD from other dementias. Subsequently, we examined the levels of serum NfL, GFAP, and p-tau217 in other dementias, further exploring the effect of combining multiple markers on the differential diagnosis of AD from other dementias. In the differential diagnosis of AD versus VaD, serum SV2A demonstrated a high diagnostic AUC, which was significantly higher than that of GFAP (*p* = 0.0087), NfL (*p* = 0.0086), and p-tau217 (*p* = 0.0010) (Additional file 1: Table S4). Serum NfL levels were significantly lower in AD than in VaD (Table 1), and the AUC for the differential diagnosis was 64.90% (Fig. 4a). Meanwhile, when combined with serum SV2A, the efficacy significantly increased to 85.6% (*p* = 0.0001) (Fig. 4d and Additional file 1: Table S4). Contrary to NfL levels, serum GFAP and p-tau217 levels were significantly higher in AD than in VaD (Table 1). When combined with serum SV2A, the AUC of GFAP for identifying AD from VaD significantly increased from 66.3 to 83.6% (*p* = 0.0007) (Fig. 4b-e), and that of p-tau217 from 63.1 to 81.9% (*p* = 0.0008) (Fig. 4c-f). The AUC of the diagnostic model combining the above four biomarkers in the differential diagnosis of AD from VaD significantly increased to 86.4% (Fig. 4g and Additional file 1: Table S4). We then combined serum SV2A with NfL, GFAP, or p-tau217 by the serial test and found that the specificity of identifying AD from VaD improved from 83.72 to 97.7%, 93.0%, or 93.0%, respectively (Table 3). On the other hand, the sensitivity values of the above diagnostic models improved to 80.5%, 86.6%, and 93.9% in the parallel test, respectively. The diagnostic model combining the above four biomarkers achieved a sensitivity of 98.2% by the parallel test and a specificity of 100.0% by the serial test (Table 3).

In the differential diagnosis of AD versus PDD, serum SV2A demonstrated a high diagnostic AUC, which was significantly higher than those of GFAP (*p* = 0.0430), NfL (*p* = 0.0009), and p-tau217 (*p* < 0.0001) (Additional file 1: Table S5). Although p-tau217 had no significant diagnostic value, the diagnostic AUC significantly increased to 85.5% when combined with serum SV2A (Fig. 4m). Serum NfL and GFAP levels were significantly higher in AD than in PDD (Table 1), and the AUCs in the differential diagnosis of AD from PDD significantly improved to 86.8% (*p* < 0.0001) (Fig. 4k) and 88.1% (*p* = 0.0001) (Fig. 4l), respectively, when combined with serum SV2A. The diagnostic model combining the above four biomarkers further improved the AUC for the differential diagnosis of AD and PDD to 89.6% (Fig. 4n), which was significantly higher than the value for the corresponding single biomarker (*p* < 0.01) (Additional file 1: Table S5). The specificity of 93.33% suggested that serum SV2A was an excellent biomarker of discriminating AD from PDD (Table 2), to improve its sensitivity, we combined serum SV2A with NfL, GFAP, or p-tau217 by the parallel test and found that the sensitivity of 64.63% increased to 83.5%, 93.3%, and 88.4%, respectively, while the sensitivity of the diagnostic model combining the four markers increased to 97.6% (Table 3).

### Serum SV2A can effectively differentiate those at high risk of AD in the cognitively unimpaired population

Serum SV2A demonstrated significant diagnostic efficacy in the early diagnosis of AD, which prompted us to further explore the ability of the biomarker to identify those at high risk of AD in cognitively unimpaired individuals. We examined serum SV2A levels in 55 cognitively unimpaired *APOE* ε4 carriers and 60 cognitively unimpaired *APOE* ε4 non-carriers by the Simoa method, which showed that serum SV2A levels were significantly lower in *APOE* ε4 carriers than in *APOE* ε4 non-carriers (*p* < 0.0001), and the difference remained statistically significant even after correcting for age and sex (*p* = 0.003). In addition, we also examined the levels of serum NfL, GFAP, and p-tau217 in the above two groups. Although the mean levels of all three biomarkers were higher in *APOE* ε4 carriers than in *APOE* ε4 non-carriers, only the increase in GFAP was statistically significant (*p* = 0.024); unfortunately, the statistical significance disappeared after correcting for age and sex (*p* = 0.052). Detailed data were shown in Table 5.

**Table 5:** Clinical and demographic features of cognitively unimpaired subjects who have undergone *APOE* testing

	APOE ε4 −/−	APOE ε4 +/−	*p*-value	*p*-value*
**No.**	60	55	NA	NA
**Sex, female/male**	35/25	32/23	0.987	NA
**Age, years (SD)**	69.23 ± 8.38	71.44 ± 6.80	0.1296	NA
**Education level, years (SD)**	12.50 ± 2.80	13.25 ± 2.34	0.125	0.184
**MMSE score, mean (SD)**	29.93 ± 0.25	29.93 ± 0.25	0.900	0.705
**MOCA score, mean (SD)**	29.93 ± 0.26	29.91 ± 0.29	0.632	0.512
**SV2A**	8088.85 ± 10222.24	2605.98 ± 2488.92	**< 0.0001**	**0.003**
**NfL**	6.82 ± 7.22	8.97 ± 13.63	0.691	0.316
**GFAP**	8.17 ± 7.61	12.90 ± 11.85	**0.024**	0.052
**p-tau217**	1.43 ± 0.49	1.60 ± 0.66	0.315	0.158

Note: The normality of the distribution of the variables was assessed by the Shapiro–Wilk test. Continuous variables were compared between two independent samples using the t-test or the Mann–Whitney U test. Categorical data such as sex was analyzed using the chi-square test. *Logistic regression was employed to compare continuous variables between different groups after adjusting for covariates such as age and sex. Abbreviations: *APOE* ε4 −/−, *APOE* ε4 non-carriers; *APOE* ε4 +/−, *APOE* ε4 carriers; GFAP, glial fibrillary acidic protein; MMSE, Mini-Mental State Examination; MoCA, Montreal Cognitive Assessment; NfL, neurofilament light; NA, not available; p-tau217, phosphorylated tau; SD, standard deviation. SEN, sensitivity; SPE, specificity; SV2A, synaptic vesicle glycoprotein 2 A

**Fig. 5:**
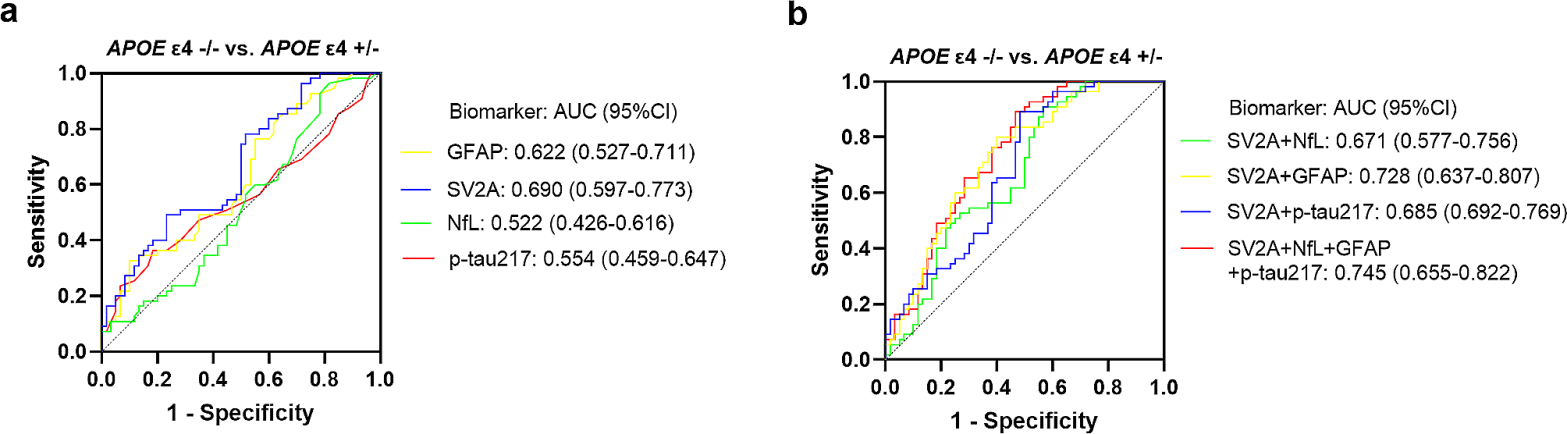
Effectiveness of serum SV2A and other biomarkers for AD risk individual identification. (**a**) Efficacy of serum SV2A, NfL, GFAP, and p-tau217 in differentiating cognitively unimpaired *APOE* ε4 carriers from cognitively unimpaired *APOE* ε4 non-carriers, respectively. (**b**) Efficacy of serum SV2A + NfL, SV2A + GFAP, SV2A + p-tau217, and SV2A + NfL + GFAP + p-tau217 in differentiating cognitively unimpaired *APOE* ε4 carriers from cognitively unimpaired *APOE* ε4 non-carriers, respectively. Abbreviations: 95% CI, 95% confidence interval; *APOE* ε4 −/−, *APOE* ε4 non-carriers; *APOE* ε4 +/−, *APOE* ε4 carriers; AUC, area under the curve; GFAP, glial fibrillary acidic protein; NfL, neurofilament light; p-tau217, phosphorylated tau; SV2A, synaptic vesicle glycoprotein 2 A; vs., versus

Diagnostic accuracy was evaluated using ROC curve analysis, which showed that serum SV2A could significantly identify *APOE* ε4 carriers from *APOE* ε4 non-carriers, with a diagnostic AUC of 69.0% (95% CI = 0.597–0.773) (Fig. 5a), which was higher than that of other three biomarkers, where the difference with NfL was significant (*p* = 0.0193) and the difference with p-tau217 was approaching significance (*p* = 0.0688) (Additional file 1: Table S6). Although NfL, p-tau217, and GFAP had poor efficacy in distinguishing cognitively unimpaired *APOE* ε4 carriers from cognitively unimpaired *APOE* ε4 non-carriers, the diagnostic AUCs of the three biomarkers significantly improved to 67.1%, 72.8%, and 68.5%, respectively, when combined with serum SV2A, and the differential diagnostic AUC of the diagnostic model combining the four biomarkers increased to 74.5%, which was significantly higher than those of the corresponding single indicator, as well as the diagnostic model combining NfL, p-tau217 and GFAP (*p* < 0.05) (Fig. 5a-b and Additional file 1: Table S6). The sensitivity of 81.82% indicated that serum SV2A was suitable for screening individuals at risk for AD. To improve the specificity of SV2A, we combined it with GFAP, which demonstrated high specificity (90.0%). The specificity of this diagnostic model increased to 91.7% by the serial test, and the specificity further increased to 98.3% when SV2A was combined with the other three biomarkers. On the other hand, the results of the parallel test showed that serum SV2A combined with GFAP and p-tau217 increased the screening sensitivity for individuals at high risk of AD to 89.1% and 92.7%, respectively, and the sensitivity of the diagnosis with the above four biomarkers combined reached 100.0% (Table 6).

**Table 6:** The efficiency of serum SV2A combined with other biomarkers in discriminating cognitively unimpaired *APOE* ε4 carriers from *APOE* ε4 non-carriers

		APOE ε4 −/− vs. APOE ε4 +/−				
		AUC (95% CI)	Cutoff (pg/mL)	SEN (%)	SPE (%)	
**SV2A**		0.690 (0.597–0.773)	≤ 4413.00	81.82	48.33	
**NfL**		0.522 (0.426–0.616)	> 1.1	96.36	18.33	
**GFAP**		0.622 (0.527–0.711)	> 16.6	32.73	90.00	
**p-tau217**		0.554 (0.459–0.647)	> 1.8	36.36	81.67	
**SV2A + NfL**		0.671 (0.577–0.756)	NA	100.0*	55.0†	
**SV2A + GFAP**		0.728 (0.637–0.807)	NA	89.1*	91.7†	
**SV2A + p-tau217**		0.685 (0.692–0.769)	NA	92.7*	90.0†	
**SV2A + p-tau217 + GFAP + NfL**		0.745 (0.655–0.822)	NA	100.0*	98.3†	

Note: We assessed the normality of the distribution of the variables by the Shapiro–Wilk test. Logistic regression was used to evaluate predictive models and receiver operating characteristic (ROC) curves constructed from the logistic scores. The Youden index was calculated to determine the best cutoff regarding sensitivity and specificity for the single indicator. The *parallel test and ^†^serial test was used to calculate the sensitivity and specificity of the combined diagnostic models, respectively. Abbreviations: 95%CI, 95% confidence interval; *APOE* ε4 −/−, *APOE* ε4 non-carriers; *APOE* ε4 +/−, *APOE* ε4 carriers; AUC, area under the curve; GFAP, glial fibrillary acidic protein; NfL, neurofilament light; p-tau217, phosphorylated tau; SEN, sensitivity; SPE, specificity; SD, standard deviation; SV2A, synaptic vesicle glycoprotein 2 A; vs., versus

The Youden index was calculated to determine the best cutoff value between *APOE* ε4 carriers and *APOE* ε4 non-carriers for each biomarker. Patients with SV2A concentrations below its cutoff value (≤ 4413.00 pg/mL) were defined as serum SV2A-positive cases. Similarly, patients with concentrations above the cutoff values (> 1.1 pg/mL for NfL, > 16.6 pg/mL for GFAP, and > 1.8 pg/mL for p-tau217) were defined as NfL, GFAP, and p-tau217–positive cases, respectively (Table 6). Then, we counted the number of SV2A-, GFAP-, NfL-, and p-tau217-positive cases in the AD high-risk group (*APOE* ε4 carriers) and found that a total of 45 cases were positive for SV2A, with a positivity rate of 81.82%, which was higher than those of GFAP (32.73%) and p-tau217 (36.36%) (Table 7). Although the positivity rate of NfL was 96.36%, the indicator with an extremely low specificity (18.33%) was not a statistically significant early warning marker of AD (Table 6). Then, we counted the number of SV2A-positive cases in GFAP-, NfL-, and p-tau217-negative cases among *APOE* ε4 carriers (≤ 1.1 pg/mL for NfL, ≤ 16.6 pg/mL for GFAP, and ≤ 1.8 pg/mL for p-tau217), and the results showed that the positivity rate of SV2A in GFAP-, NfL-, and p-tau217-negative cases reached 83.78%, 100.00%, and 88.57%, respectively. Finally, the positivity rate significantly increased to 89.09%, 92.73%, and 100.00% among *APOE* ε4 carriers when serum SV2A-positive cases were counted together with positive cases of GFAP, p-tau217 and NfL, respectively (Table 7).

**Table 7:** Positive rates of SV2A in *APOE* ε4 carriers who were negative for other biomarkers

	No.	Positive rate (%)
**SV2A (+) /** ***APOE*** **ε4 +/−**	45/55	81.82
**NfL (+) /** ***APOE*** **ε4 +/−**	53/55	96.36
**GFAP (+) /** ***APOE*** **ε4 +/−**	18/55	32.73
**p-tau217 (+) /** ***APOE*** **ε4 +/−**	20/55	36.36
**SV2A (+) or GFAP (+) /** ***APOE*** **ε4 +/−**	49/55	89.09
**SV2A (+) or p-tau217 (+) /** ***APOE*** **ε4 +/−**	51/55	92.73
**SV2A (+) or NfL (+) /** ***APOE*** **ε4 +/−**	55/55	100.00
**SV2A (+) / NfL (−)-** ***APOE*** **ε4 +/−**	2/2	100.00
**SV2A (+) / GFAP (−)-** ***APOE*** **ε4 +/−**	31/37	83.78
**SV2A (+) / p-tau217 (−)-** ***APOE*** **ε4 +/−**	31/35	88.57

Note: The Youden index was calculated to determine the best cutoff value. Abbreviations: *APOE* ε4 +/−, *APOE* ε4 carriers; GFAP(−)-*APOE* ε4 +/−, serum GFAP-negative patients (≤ 16.6 pg/mL in *APOE* ε4 +/−); GFAP(+), serum GFAP-positive patients (> 16.6 pg/mL in *APOE* ε4 +/−); GFAP, glial fibrillary acidic protein; NfL(−)-*APOE* ε4 +/−, serum NfL-negative patients (≤ 1.1 pg/mL in *APOE* ε4 +/−); NfL(+), serum NfL-positive patients (> 1.1 pg/mL in *APOE* ε4 +/−); NfL, neurofilament light; p-tau217(−)-*APOE* ε4 +/−, serum p-tau217-negative patients (≤ 1.8 pg/mL in *APOE* ε4 +/−); p-tau217(+), serum p-tau217-positive patients (> 1.8 pg/mL in *APOE* ε4 +/−); p-tau217, phosphorylated tau; SV2A (+), serum SV2A-positive patients (≤ 4413.00 pg/mL in *APOE* ε4 +/−); SV2A, synaptic vesicle glycoprotein 2 A

### SV2A affect Aβ pathology in APP/PS1 mice

**Fig. 6:**
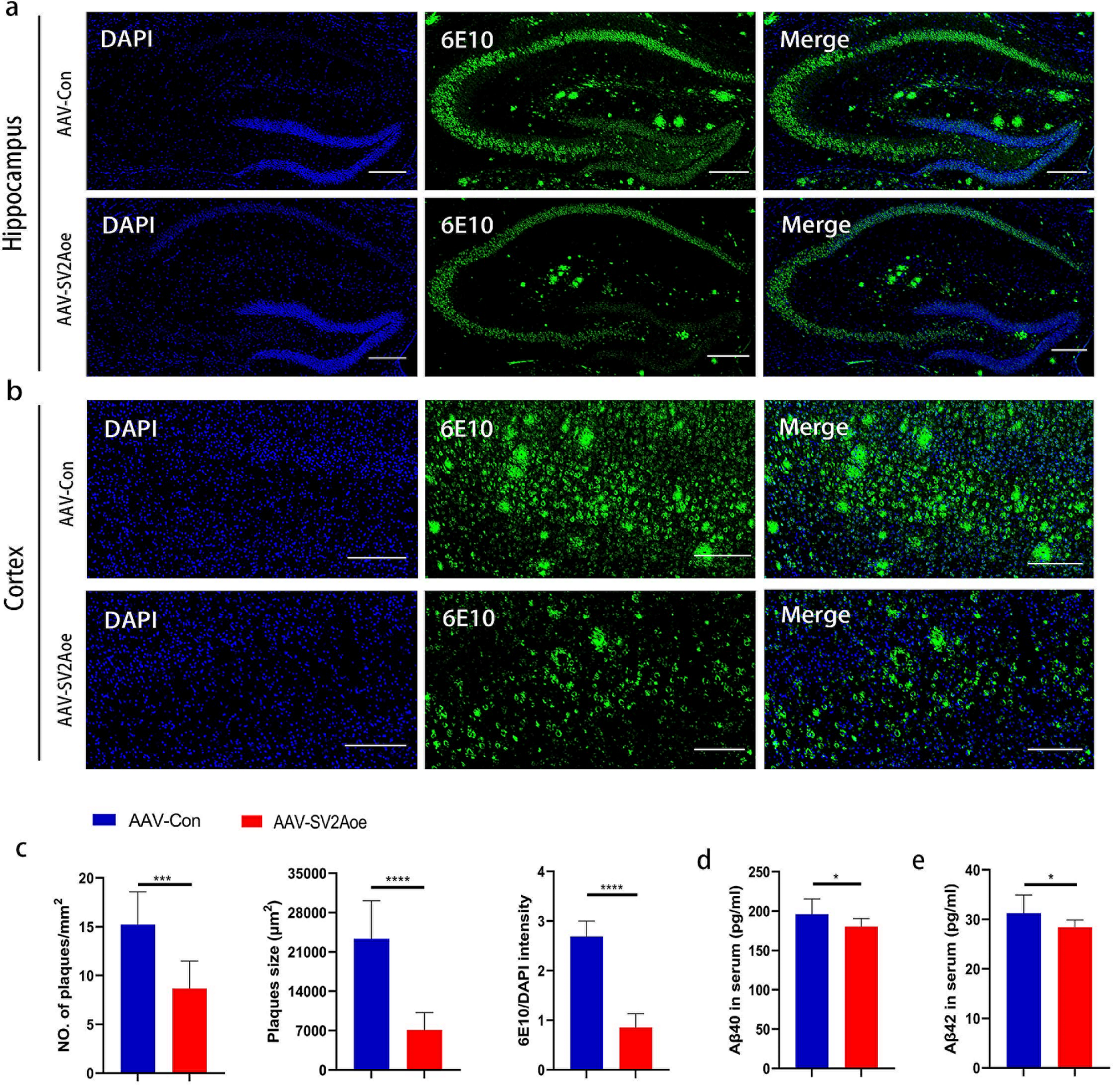
Effect of SV2A on Aβ pathology in APP/PS1 mice. (**a**) Fluorescence intensity of 6E10 in hippocampal regions of SV2A overexpressing APP/PS1 mice and control APP/PS1 mice. (**b**) Fluorescence intensity of 6E10 in cortical regions of SV2A overexpressing APP/PS1 mice and control APP/PS1 mice. (**c**) Quantification of amyloid plaques per mm^2^ area, amyloid plaque size (µm^2^), and the ratio of fluorescence intensity of 6E10 to DAPI in brain sections. AAV-SV2Aoe APP/PS1 mice group, *n* = 3; AAV-Con APP/PS1 mice group, *n* = 3; 3 fields of view per group. (**d**) The serum levels of Aβ40 in SV2A overexpressing APP/PS1 mice and control mice. (**e**) The serum levels of Aβ42 in SV2A overexpressing APP/PS1 mice and control mice. Scale bar: 50 μm. Data were presented as mean ± SD. All dot plots: t-test. ^*^p < 0.05, ^**^p < 0.01, ^***^p < 0.001, ^****^p < 0.0001

Aβ pathology was recognized as the key factor in the development of AD. To assess the value of SV2A in predicting Aβ pathology, SV2A overexpressing APP/PS1 mice and control APP/PS1 mice were constructed by brain stereotactic injection technique, respectively. Firstly, immunofluorescence staining with 6E10 antibody, which recognized the first 16 amino acids of the Aβ sequence, was conducted to identify Aβ plaques in brain sections of above two groups (Fig. 6a-b). The results indicated that the number and size of Aβ plaques and 6E10 antibody staining intensity in the brain tissues of SV2A overexpressing APP/PS1 mice was significantly reduced compared with control mice (Fig. 6c). Meanwhile, levels of metabolites of Aβ precursor protein (APP) in the serum of the above two groups of mice were measured by ELISA, and the results showed that the serum levels of both Aβ40 (Fig. 6d) and Aβ42 (Fig. 6e) were significantly reduced in SV2A overexpressing APP/PS1 mice compared to control mice. Therefore, the above results revealed that SV2A could significantly inhibit Aβ pathology in APP/PS1 mice.

## Discussion

Currently, AD has emerged as one of the greatest challenges threatening human health and affecting tens of millions of people worldwide [29]. Given the limited efficacy of pharmacological treatments in the dementia stage of AD, early intervention of AD has already been an important strategy to delay the disease progression, which relies on the early screening of AD [30]. Therefore, an excellent early warning and diagnostic biomarker is urgently needed [31]. To our knowledge, this is the first study reporting that serum SV2A could be an ideal biomarker for early screening of AD.

SV2A has been recognized as the first marker reflecting synaptic density [14, 17, 32], with ensuing evidence revealing the causal role of synapse loss and damage in AD [33, 34]. Previous studies have shown a significant positive correlation between performance in cognitive tests and ^11^C-UCB-J binding in the hippocampus [35], which suggested that SV2A by PET could be a promising biomarker of synaptic density for tracking AD progression [15, 36, 37]. As indirect evidence of histopathological changes in the brain, CSF SV2A levels in this study significantly correlated with the cognitive performance of patients with AD and progressively declined with AD progression, which reflects the role of CSF SV2A in the monitoring of AD progression. Although CSF SV2A demonstrated high diagnostic performance in the dementia phase of AD, its remarkable diagnostic efficacy in the early stages of AD was of great interest. However, given the invasive nature of CSF sampling, we subsequently focused on the role of blood SV2A in the early diagnosis of AD. It is worth emphasizing that the significant correlation between serum and CSF SV2A supported the idea that this assay specifically measured brain-derived SV2A, which also indirectly reflected pathological changes in the brain tissue of patients with AD.

In this study, serum SV2A levels were significantly lower in the aMCI group than in the control group and demonstrated significant efficacy in the diagnosis of aMCI, which was consistent with what we found in CSF, as well as the results of the PET imaging that reported by other scholars. For example, PET studies have shown significant reductions of SV2A binding ([^11^C]UCB-J) across the majority of neocortical regions in the aMCI group compared with the cognitively normal group [35, 38]. Briefly, the above imaging results and CSF and serum findings suggested that the level of SV2A was decreasing in aMCI. To further validate the significance of SV2A in the early diagnosis of AD, we simultaneously examined the levels of three core AD biomarkers (NfL, GFAP, and p-tau217) by the Simoa method. Although these non-synaptic biomarkers demonstrated some effectiveness in the diagnosis of aMCI, SV2A-positive rates exceeded 90% in aMCI cases that were negative for NfL, GFAP, or p-tau217, which suggested that serum SV2A was more suitable as a screening indicator in the early stage of AD. The high sensitivity (97.80%) made SV2A suitable for screening early AD, which inevitably led to the low specificity. To compensate for this shortcoming, we combined serum SV2A with serum p-tau217, which has high specificity, by the serial test, which showed that the diagnostic specificity of the diagnostic model for aMCI increased to 95.1%, and the diagnostic AUC significantly increased to nearly 90.0%.

Given the high efficacy of serum SV2A in early screening of aMCI, we next explored its value in distinguishing those at high risk of AD in a cognitively unimpaired population. *APOE* ε4 has been considered the strongest genetic risk factor associated with sporadic AD by multiple large-scale genome-wide association studies (GWAS) and GWAS meta-analyses [39]. Relative to the most common *APOE* ε3/ε3 genotype, possessing one *APOE* ε4 allele increases the risk of AD development by approximately 3.7 times, and being homozygous for the *APOE* ε4 allele increases the risk up to 12 times [40]. In addition, *APOE* ε4 carriers have more amyloid deposition, earlier disease onset, and progress more rapidly once the symptomatic phase initiates [41]. In this study, serum SV2A levels were significantly lower in cognitively unimpaired *APOE* ε4 carriers than in cognitively unimpaired non-carriers before and after adjusting for age and sex, suggesting that serum SV2A levels had already decreased during the preclinical phase of AD. Previous studies have concluded that the presence of *APOE* ε4 was associated with increased synaptic degeneration [42]. For instance, Love S et al. found that the levels of the synaptic markers synaptophysin, syntaxin 1 and postsynaptic density protein 95 (PSD95) were reduced in the brains of *APOE* ε4 carriers [43], while another study demonstrated that reduced expression of synaptic proteins [44] and impaired synaptic transmission [45] have all been observed in *APOE* ε4-targeted replacement (TR) mice when compared with *APOE* ε3-TR mice. In addition, *APOE* ε4 has a toxic effect on synapse-related pathways [46]. Since SV2A is widely expressed at synapses [35] and it reflects synaptic density in the brain [14], we therefore suggest that it is the damage to synapses by *APOE* that leads to the reduction of SV2A.

Although the mean levels of GFAP, p-tau217, and NfL increased in cognitively unimpaired *APOE* ε4 carriers, the increases in p-tau217 and NfL levels were not statistically significant, and that of GFAP lost statistical significance after correcting for age and sex. Regarding the diagnostic efficacy of differentiating high-risk populations from cognitively normal individuals, serum SV2A had the highest diagnostic performance of all biomarkers, and in particular, the high sensitivity made it more suitable as a screening marker for individuals with a high risk of AD. Specifically, the positivity rate of SV2A in cognitively unimpaired *APOE* ε4 carriers was significantly higher than those of GFAP and p-tau217, and SV2A was positive in more than 80% of cognitively unimpaired *APOE* ε4 carriers who were negative for GFAP or p-tau217. Therefore, the above data supported the conclusion that the efficacy of serum SV2A in identifying individuals at high risk of AD in a cognitively normal population was superior to those of other markers tested. As a potential screening indicator for those at high risk of AD, although serum SV2A had a high sensitivity, it did not perform as well in terms of specificity. To compensate for this shortcoming, we analyzed serum SV2A in combination with serum GFAP, a biomarker with high specificity, and the serial test results showed that the specificity of this diagnostic model for populations at risk of AD significantly increased to 91.7%. Of course, the combination of other biomarkers based on multiple pathologic mechanisms would also enhance the efficacy of screening for populations at high risk for AD. Just as we found that when combined with serum SV2A, the ability of non-synaptic biomarker NfL and p-tau217 to discriminate cognitively unimpaired *APOE* ε4 carriers from non-carriers was significantly enhanced, and the positivity in cognitively unimpaired *APOE* ε4 carriers was also improved.

In addition to the outstanding diagnostic performance in the early and preclinical stages of AD, another point of interest was the great potential demonstrated by SV2A to differentiate AD dementia from non-AD dementia. Firstly, we must emphasize the excellent performance of SV2A in the diagnosis of AD, of which the AUCs of the CSF and serum SV2A were 91.37% and 86.6%, respectively, and the decrease of CSF and serum SV2A levels in patients with AD was consistent with the trend in SV2A PET [17]. However, SV2A did not appear to be AD-specific from our findings, as evidenced by the fact that SV2A levels in both CSF and serum were lower in the VaD and PDD groups than in the control group, although the reduction was not statistically significant. The previous SV2A PET had shown significantly lower ^11^C-UCB-J in the cortical regions and subcortical regions of the PD patients compared to control subjects, which in combination with other studies suggest that PET SV2A also had diagnostic value in other dementias [47–49]. However, the characteristics of non-AD specificity did not overshadow the excellent performance of SV2A in the differential diagnosis of AD from VaD and PDD. First, the mean levels of CSF and serum SV2A in AD were the lowest in the three dementias. Second, SV2A demonstrated high diagnostic efficacy in the differential diagnosis of AD dementia from non-AD dementia, of which the AUC of CSF SV2A for distinguishing AD from VaD and PDD was > 90%. Although the AUC of serum SV2A for distinguishing AD dementia from non-AD dementia was not as prominent as that of CSF SV2A, it was significantly higher than those of serum GFAP, NfL, and p-tau217. Finally, the high specificity of SV2A both in CSF and serum made it superior in the differential diagnosis of AD dementia from non-AD dementia, of which the specificity of serum SV2A was significantly higher than that of the other three biomarkers tested.

In the above study we focused on the value of serum SV2A for aMCI, AD patients and those at high risk of AD. Currently, Aβ pathology was recognized as the core mechanism in the development of AD [50, 51]. To assess the predictive value of SV2A on Aβ pathology, APP/PS1 mice overexpressing SV2A were successfully constructed by brain stereotactic injection in the present study, followed by observation of Aβ pathology in brain tissues of the model mice. The results showed that the number and size of Aβ plaques in the hippocampus and cortical regions of SV2A overexpressing APP/PS1 mice were significantly reduced compared with those of the control group, which also preliminarily revealed the negative correlation between SV2A and Aβ pathology in APP/PS1 mice. Recently, several studies have reported the role of SV2A on Aβ pathology. For example, Kasatkina et al. suggested that levetiracetam, a specific modulator of SV2A, could upregulate the level of SV2A in hippocampus region, which in turn reduced the level of Aβ precursor protein (APP) and restricted synaptic activity dependent Aβ accumulation in the rat model of neuroinflammation [52]. Kong et al. found that the level of Aβ was significantly increased in APPswe293T cells with down-regulated expression of SV2A as well as in brain tissues of SV2A knockout mice, and significantly decreased in APPswe293T cells with overexpression of SV2A, as compared to the corresponding controls [53]. Given that Aβ is the product of APP amyloid degradation, the potential mechanism by which SV2A affects Aβ may be the participation of SV2A in the amyloid metabolism of APP. In our experiment, the serum levels of APP amyloid degradation products in the above two groups of APP/PS1 mice were also measured, and the results showed that the serum levels of Aβ42 and Aβ40 were significantly reduced in SV2A overexpressing APP/PS1 mice compared with the control group. Therefore, it was hypothesized that SV2A inhibited Aβ pathology by regulating the APP amyloid degradation pathway.

This study innovatively explored the role of serum SV2A in patients with AD by the Simoa, which will promote the application of the marker in more fields, including early screening, differential diagnosis, and treatment monitoring of AD, and provide a new technical tool for the study of SV2A in other neurodegenerative diseases. Given the high cost of PET and the invasive nature of CSF testing, the development of blood markers for AD will be a key focus of future research. To our best knowledge, no study of blood SV2A has been described, and the role of serum SV2A in AD observed in our study may end the long history of the absence of blood markers reflecting synaptic pathology [54]. In addition, the average SV2A level was higher than that of other markers, which made it easier to detect in the early stages of AD, particularly during the preclinical phase of AD.

## Limitations

In this study, serum SV2A was found to be an ideal early warning and diagnosis biomarker for AD, as well as monitoring the progress of AD. Another aim of this work was to accurately diagnose and conduct differential diagnosis of dementia using specific blood biomarkers, demographics, and cognitive scores, thus providing clinical insights into the use of blood biomarkers in the diagnosis of AD.

As a limitation, this study did not include replication cohorts of CSF and blood biomarkers obtained under different platform assays. In addition, the study did not involve longitudinal follow-up, thus, stable MCI and MCI that progresses to dementia could not be discriminated. On the other hand, ethylenediaminetetraacetic acid plasma samples are recommended for the Simoa assay of Aβ40 and Aβ42 [55], and the levels in our serum samples were extremely low or not detected, therefore, the results of Aβ40 and Aβ42 could not be analyzed. Multicenter and longitudinal studies are needed to confirm and improve the generalizability of the results of this study.

## Conclusions

For the first time, we successfully detected SV2A in blood by the Simoa method. More importantly, the high efficacy of serum SV2A in the early screening of AD, coupled with the easily accessible and non-invasive nature of the blood test, makes serum SV2A an excellent biomarker for healthy populations screening.

### Electronic supplementary material

Below is the link to the electronic supplementary material.

Supplementary Material 1

## Data Availability

The datasets used during the current study are available from the corresponding author on reasonable request.

## Abbreviations

AD: Alzheimer’s disease
aMCI: amnestic mild cognitive impairment
*APOE* ε4: apolipoprotein E ε4
GFAP: glial fibrillary acidic protein
MMSE: Mini-Mental State Examination
MoCA: Montreal Cognitive Assessment
NfL: neurofilament light
PDD: Parkinson’s disease dementia
p-tau217: phosphorylated tau
SV2A: synaptic vesicle glycoprotein 2 A
VaD: vascular dementia
