# Supplementary material for: Synaptic vesicle glycoprotein 2 A in serum is an ideal biomarker for early diagnosis of Alzheimer’s disease
Source: Alzheimers Res Ther. 2024 Apr 13;16:82. doi: 10.1186/s13195-024-01440-9 (PMC11015666; doi:10.1186/s13195-024-01440-9)
Supplement: Supplementary file 1 — Supplementary Material 1 [file 13195_2024_1440_MOESM1_ESM.docx]

**Synaptic vesicle glycoprotein 2A in serum is an ideal biomarker for early diagnosis of Alzheimer’s disease**

**Additional file 1**

**Supplementary Fig. 1** Standard curves for biomarkers.

**Supplementary Table 1** Comparison of the AUCs for distinguishing Con and aMCI between the combined diagnostic models and single blood biomarkers.

**Supplementary Table 2** Comparison of the AUCs for distinguishing aMCI and AD between the combined diagnostic models and single blood biomarkers.

**Supplementary Table 3** Comparison of the AUCs for distinguishing Con and AD between the combined diagnostic models and single blood biomarkers.

**Supplementary Table 4** Comparison of the AUCs for distinguishing VaD and AD between the combined diagnostic models and single blood biomarkers.

**Supplementary Table 5** Comparison of the AUCs for distinguishing PDD and AD between the combined diagnostic models and single blood biomarkers.

**Supplementary Table 6** Comparison of the AUCs for distinguishing *APOE* ε4 −/− and *APOE* ε4 +/− between the combined diagnostic models and single blood biomarkers.


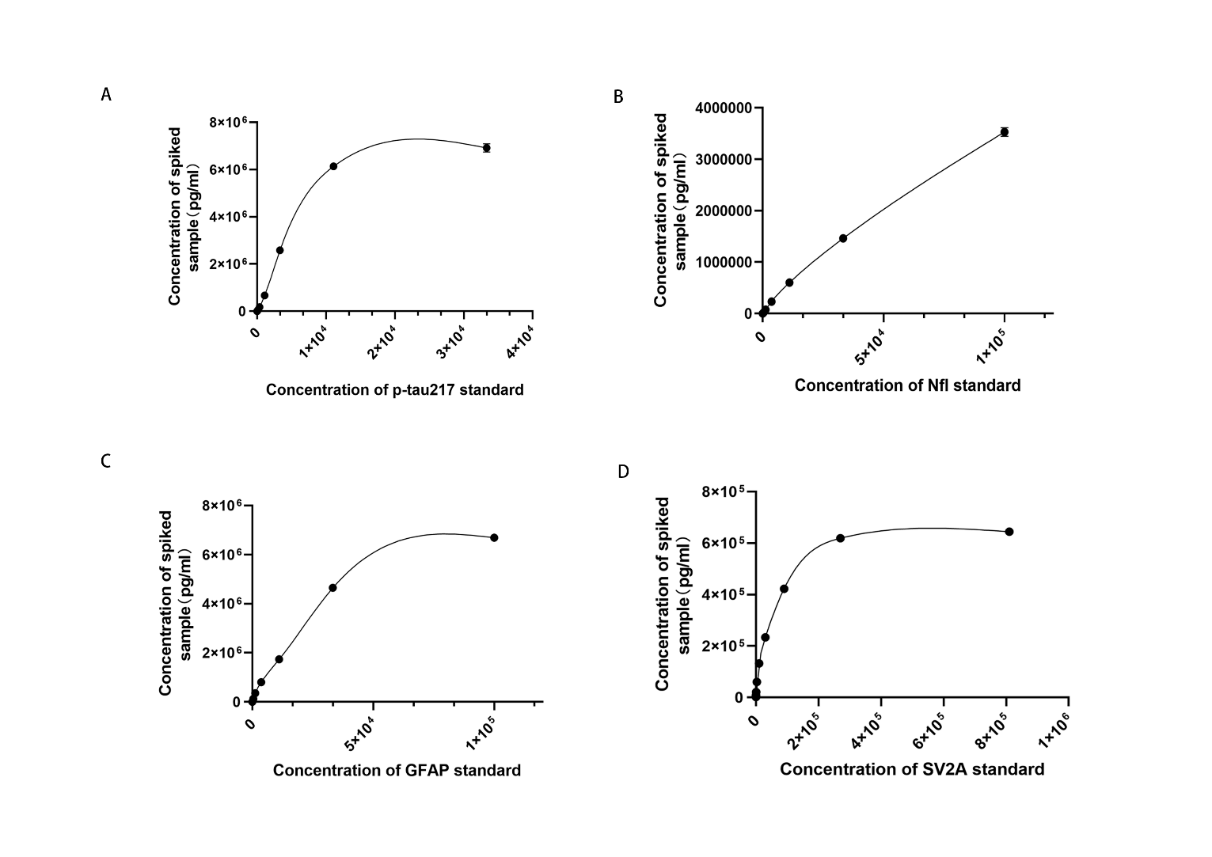


**Supplementary Fig. 1 Standard curves for biomarkers.** Standard curves for p-tau217 (A), NfL (B), GFAP (C) and SV2A (D). Abbreviations: GFAP, glial fibrillary acidic protein; NfL, neurofilament light; p-tau217, phosphorylated tau; SV2A, synaptic vesicle glycoprotein 2A.

**Establishment of standard curves for each biomarker**

Standards of SV2A, p-tau217, NfL and GFAP were diluted with commercial serum matrix (TRINA, T3/T4 Free Control). Each diluted sample was measured as steps: (1) Load 25 μL sample into an incubation tube and add Reagent 1 (mainly comprised 0.1 mg/mL magnetic beads coated with capture antibodies and protecting reagents), followed by a quick mixing by the machine. (2) After 6-minutes incubation, Reagent 2 (comprised of detection antibodies labeled with single-molecule imagine fluorophores) was added, mixed and incubated for 4 minutes under 40°C. (3) Magnetic beads in the mixtures were absorbed onto the surface of the channel in the flow cell by a permanent magnet. Unlabeled fluorophores were removed by a gentle washing flow of wash buffer and fluorescent images were then taken with an integrated fluorescent microscope. (4) Standard curves for each serum biomarkers are built with Prism 9 software (GraphPad, San Diego, CA, USA). Each sample was tested 3 times and the mean, standard deviation and coefficient of variation were calculated. The following equation was used to calculate LOD (limit of detection): LOD = 2δ/s (δ: standard deviation of non-spiked sample; s: slope of the standard curve).

**Development and performance of CSF and serum single‑molecule array (Simoa)**

The simoa assay demonstrated high analytical performance. The SV2A simoa assay covered a range from 0 ng/ml to 270 ng/ml and the intra-assay CV was 1.6-9.9%; we calculated the LOD (limit of detection) of 15pg/ml for SV2A assay, with all samples measured above the assay LOD. The GFAP simoa assay covered a range from 0 ng/ml to 100 ng/ml and the intra-assay CV was 2.2%-9.0%; we calculated the LOD of 0.61 pg/ml for GFAP assay, with 654 out of 666 (98.2%) samples measured above the assay LOD and the 12 samples measuring below the LOD included Control (n = 4), PDD (n = 1) and cognitively unimpaired *APOE* ε4 non-carriers (n = 7). The p-tau217 simoa assay covered a range from 0 ng/ml to 10 ng/ml and the intra-assay CV was 1.4%-8.4%; we calculated the LOD of 0.096 pg/ml for p-tau217 assay, with 665 out of 666 (99.8%) samples measured above the assay LOD, and the sample measuring below the LOD was Control (n = 1). The NfL simoa assay covered a range from 0 ng/ml to 100 ng/ml and the intra-assay CV was 1.6%-9.3%; we calculated the LOD of 1.53 pg/ml for NfL assay, with 638 out of 666 (95.8%) samples measured above the assay LOD, and the 28 samples measuring below the LOD included Control (n = 4), aMCI (n = 1), cognitively unimpaired *APOE* ε4 non-carriers (n = 13) and cognitively unimpaired *APOE* ε4 carriers (n = 10). All the above samples below LOD were serum samples. No cross-reaction with human albumin or immunoglobulin G was observed.

**Supplementary Table 1 Comparison of the AUCs for distinguishing Con and aMCI between the combined diagnostic models and single blood biomarkers**

| **Con vs. aMCI** | **SV2A (0.741)** | **GFAP (0.777)** | **p-tau217 (0.832)** | **NfL (0.590)** | **GFAP + p-tau217 + NfL (0.871)** |
| --- | --- | --- | --- | --- | --- |
| **SV2A (0.741)** | **−** | 0.4794 | 0.0539 | **0.0137** | **−** |
| **SV2A + GFAP (0.865)** | **0.0001** | **0.0014** | **−** | **−** | **−** |
| **SV2A + p-tau217 (0.891)** | **<0.0001** | **−** | **0.0027** | **−** | **−** |
| **SV2A + NfL (0.771)** | 0.2145 | **−** | **−** | **0.0001** | **−** |
| **SV2A + GFAP + p-tau217 + NfL (0.918)** | **<0.0001** | **<0.0001** | **0.0006** | **<0.0001** | **0.0045** |

Note: Logistic regression was used to evaluate predictive models and receiver operating characteristic (ROC) curves constructed from the logistic scores. The Delong test was used to compare the AUCs of the diagnostic models. Abbreviations: Con, healthy control; aMCI, amnestic mild cognitive impairment; GFAP, glial fibrillary acidic protein; NfL, neurofilament light; p-tau217, phosphorylated tau; SV2A, synaptic vesicle glycoprotein 2A; vs., versus.

**Supplementary Table 2 Comparison of the AUCs for distinguishing aMCI and AD between the combined diagnostic models and single blood biomarkers**

| **aMCI vs. AD** | **SV2A (0.702)** | **GFAP (0.663)** | **p-tau217 (0.561)** | **NfL (0.564)** | **GFAP + p-tau217 + NfL (0.683)** |
| --- | --- | --- | --- | --- | --- |
| **SV2A (0.702)** | **−** | 0.4528 | **0.0096** | **0.0159** | **−** |
| **SV2A + GFAP (0.764)** | **0.0172** | **0.0019** | **−** | **−** | **−** |
| **SV2A + p-tau217 (0.727)** | 0.2289 | **−** | **0.0001** | **−** | **−** |
| **SV2A + NfL (0.734)** | 0.1184 | **−** | **−** | **0.0001** | **−** |
| **SV2A + GFAP + p-tau217 + NfL (0.790)** | **0.0019** | **0.0002** | **<0.0001** | **<0.0001** | **0.0007** |

Note: Logistic regression was used to evaluate the predictive models and receiver operating characteristic (ROC) curves constructed from the logistic scores. The Delong test was used to compare the AUCs of the diagnostic models. Abbreviations: aMCI, amnestic mild cognitive impairment; AD, Alzheimer’s disease; GFAP, glial fibrillary acidic protein; NfL, neurofilament light; p-tau217, phosphorylated tau; SV2A, synaptic vesicle glycoprotein 2A; vs., versus.

**Supplementary Table 3 Comparison of the AUCs for distinguishing Con and AD between the combined diagnostic models and single blood biomarkers**

| **Con vs. AD** | **SV2A (0.866)** | **GFAP (0.859)** | **p-tau217 (0.862)** | **NfL (0.645)** | **GFAP + p-tau217 + NfL (0.922)** |
| --- | --- | --- | --- | --- | --- |
| **SV2A (0.866)** | **−** | 0.8120 | 0.8849 | **<0.0001** | **−** |
| **SV2A + GFAP (0.937)** | **0.0004** | **<0.0001** | **−** | **−** | **−** |
| **SV2A + p-tau217 (0.942)** | **0.0003** | **−** | **<0.0001** | **−** | **−** |
| **SV2A + NfL (0.883)** | 0.3122 | **−** | **−** | **<0.0001** | **−** |
| **SV2A + GFAP + p-tau217 + NfL (0.962)** | **<0.0001** | **<0.0001** | **<0.0001** | **<0.0001** | **0.0001** |

Note: Logistic regression was used to evaluate predictive models and receiver operating characteristic (ROC) curves constructed from the logistic scores. The Delong test was used to compare the AUCs of the diagnostic models. Abbreviations: AD, Alzheimer’s disease; Con, healthy control; GFAP, glial fibrillary acidic protein; NfL, neurofilament light; p-tau217, phosphorylated tau; SV2A, synaptic vesicle glycoprotein 2A; vs., versus.

**Supplementary Table 4 Comparison of the AUCs for distinguishing VaD and AD between the combined diagnostic models and single blood biomarkers**

| **VaD vs. AD** | **SV2A (0.823)** | **GFAP (0.663)** | **p-tau217 (0.631)** | **NfL (0.649)** | **GFAP + p-tau217 + NfL (0.734)** |
| --- | --- | --- | --- | --- | --- |
| **SV2A (0.823)** | − | **0.0087** | **0.0010** | **0.0086** | − |
| **SV2A + GFAP (0.836)** | 0.4742 | **0.0007** | − | − | − |
| **SV2A + p-tau217 (0.819)** | 0.4481 | − | **0.0008** | − | − |
| **SV2A + NfL (0.856)** | 0.0610 | − | − | **0.0001** | − |
| **SV2A + GFAP + p-tau217 + NfL (0.864)** | 0.1066 | **<0.0001** | **<0.0001** | **0.0002** | **0.0022** |

Note: Logistic regression was used to evaluate the predictive models and receiver-operating characteristic (ROC) curves constructed from the logistic scores. The Delong test was used to compare the AUCs of the diagnostic models. Abbreviations: AD, Alzheimer’s disease; GFAP, glial fibrillary acidic protein; NfL, neurofilament light; p-tau217, phosphorylated tau; SV2A, synaptic vesicle glycoprotein 2A; VaD, Vascular dementia; vs., versus.

**Supplementary Table 5 Comparison of the AUCs for distinguishing PDD and AD between the combined diagnostic models and single blood biomarkers**

| **PDD vs. AD** | **SV2A (0.848)** | **GFAP (0.725)** | **p-tau217 (0.501)** | **NfL (0.679)** | **GFAP + p-tau217 + NfL (0.772)** |
| --- | --- | --- | --- | --- | --- |
| **SV2A (0.848)** | − | **0.0430** | **<0.0001** | **0.0009** | − |
| **SV2A + GFAP (0.881)** | 0.1758 | **0.0001** | − | − | − |
| **SV2A + p-tau217 (0.855)** | 0.3286 | − | **<0.0001** | − | − |
| **SV2A + NfL (0.868)** | 0.3981 | − | − | **<0.0001** | − |
| **SV2A + GFAP + p-tau217 + NfL (0.896)** | 0.0616 | **0.0003** | **<0.0001** | **<0.0001** | **0.0005** |

Note: Logistic regression was used to evaluate the predictive models and receiver operating characteristic (ROC) curves constructed from the logistic scores. The Delong test was used to compare the AUCs of the diagnostic models. Abbreviations: AD, Alzheimer’s disease; GFAP, glial fibrillary acidic protein; NfL, neurofilament light; PDD, Parkinson’s disease dementia; p-tau217, phosphorylated tau; SV2A, synaptic vesicle glycoprotein 2A; vs., versus.

**Supplementary Table 6 Comparison of the AUCs for distinguishing *APOE* ε4 −/− and *APOE* ε4 +/− between the combined diagnostic models and single blood biomarkers**

| ***APOE* ε4 −/− vs. *APOE* ε4 +/−** | **SV2A (0.690)** | **GFAP (0.622)** | **p-tau217 (0.554)** | **NfL (0.522)** | **GFAP + p-tau217 + NfL (0.646)** |
| --- | --- | --- | --- | --- | --- |
| **SV2A (0.690)** | **−** | 0.3414 | 0.0688 | **0.0193** | **−** |
| **SV2A + GFAP (0.728)** | 0.2998 | **0.0180** | **−** | **−** | **−** |
| **SV2A + p-tau217 (0.685)** | 0.8935 | **−** | **0.0136** | **−** | **−** |
| **SV2A + NfL (0.671)** | 0.4340 | **−** | **−** | **0.0151** | **−** |
| **SV2A + GFAP + p-tau217 + NfL (0.745)** | 0.1738 | **0.0174** | **0.0007** | **0.0001** | **0.0135** |

Note: Logistic regression was used to evaluate the predictive models and receiver operating characteristic (ROC) curves constructed from the logistic scores. The Delong test was used to compare the AUCs of the diagnostic models. Abbreviations: *APOE* ε4 −/−, *APOE* ε4 non-carriers; *APOE* ε4 +/−, *APOE* ε4 carriers; GFAP, glial fibrillary acidic protein; NfL, neurofilament light; p-tau217, phosphorylated tau; SV2A, synaptic vesicle glycoprotein 2A; vs., versus.
